# Assessing Mitochondrial DNA Variation and Copy Number in Lymphocytes of ~2,000 Sardinians Using Tailored Sequencing Analysis Tools

**DOI:** 10.1371/journal.pgen.1005306

**Published:** 2015-07-14

**Authors:** Jun Ding, Carlo Sidore, Thomas J. Butler, Mary Kate Wing, Yong Qian, Osorio Meirelles, Fabio Busonero, Lam C. Tsoi, Andrea Maschio, Andrea Angius, Hyun Min Kang, Ramaiah Nagaraja, Francesco Cucca, Gonçalo R. Abecasis, David Schlessinger

**Affiliations:** 1 Laboratory of Genetics, National Institute on Aging, NIH, Baltimore, Maryland; 2 Department of Biostatistics and Center for Statistical Genetics, University of Michigan, Ann Arbor, Michigan; 3 Istituto di Ricerca Genetica e Biomedica, Consiglio Nazionale delle Ricerche, Monserrato, Cagliari, Italy; 4 Università degli Studi di Sassari, Sassari, Italy; Georgia Institute of Technology, UNITED STATES

## Abstract

DNA sequencing identifies common and rare genetic variants for association studies, but studies typically focus on variants in nuclear DNA and ignore the mitochondrial genome. In fact, analyzing variants in mitochondrial DNA (mtDNA) sequences presents special problems, which we resolve here with a general solution for the analysis of mtDNA in next-generation sequencing studies. The new program package comprises 1) an algorithm designed to identify mtDNA variants (i.e., homoplasmies and heteroplasmies), incorporating sequencing error rates at each base in a likelihood calculation and allowing allele fractions at a variant site to differ across individuals; and 2) an estimation of mtDNA copy number in a cell directly from whole-genome sequencing data. We also apply the methods to DNA sequence from lymphocytes of ~2,000 SardiNIA Project participants. As expected, mothers and offspring share all homoplasmies but a lesser proportion of heteroplasmies. Both homoplasmies and heteroplasmies show 5-fold higher transition/transversion ratios than variants in nuclear DNA. Also, heteroplasmy increases with age, though on average only ~1 heteroplasmy reaches the 4% level between ages 20 and 90. In addition, we find that mtDNA copy number averages ~110 copies/lymphocyte and is ~54% heritable, implying substantial genetic regulation of the level of mtDNA. Copy numbers also decrease modestly but significantly with age, and females on average have significantly more copies than males. The mtDNA copy numbers are significantly associated with waist circumference (p-value = 0.0031) and waist-hip ratio (p-value = 2.4×10^-5^), but not with body mass index, indicating an association with central fat distribution. To our knowledge, this is the largest population analysis to date of mtDNA dynamics, revealing the age-imposed increase in heteroplasmy, the relatively high heritability of copy number, and the association of copy number with metabolic traits.

## Author Summary

We present a new program that provides a general solution for the analysis of variation of mtDNA (the small circular genome in mitochondria, separate from the DNA in the nucleus). This is needed because many large-scale genetic studies are using new DNA sequencing technologies to help assess genetic variation and its effects on disease, but the mitochondrial genome is often ignored because it exists in many copies in a cell, complicating analyses. Our approach both identifies variants on mitochondrial genome and estimates mtDNA copy number. Applying the programs to DNA sequence from ~2,000 SardiNIA project participants, we show that heteroplasmies (mtDNA variants with more than one allele at a DNA site) increase with age, and that copy number is relatively highly heritable and is correlated with metabolic traits, particularly central fat levels. The program package can facilitate comprehensive mtDNA analysis from any whole-genome sequencing data, with an increase in the understanding of mtDNA dynamics and its potential role in aging and metabolism.

## Introduction

As the “cellular power plant”, each mitochondrion encodes some of its constituent proteins in resident mitochondrial DNA (mtDNA). Human mtDNA is a circular molecule of 16,569 bases, and mutations that have become fixed in the sequence of every mtDNA may cause several genetic diseases. Accumulation of variants during growth has been suggested to have an important role in aging and cancer[1–3]. However, although the degree to which mtDNA varies heritably and somatically has been much discussed, it has not been analyzed on a population basis.

Modern high-throughput sequencing facilitates systematic identification of common and rare DNA variants, including many associated with complex diseases and quantitative traits[4,5]. To extend comparable sequence analysis to mtDNA, an important step in the analysis pipeline must be modified. Variant identification for nuclear DNA using sequencing data has been greatly refined, typically using a likelihood-based model to combine information from sequence reads and predict the genotype with the highest posterior probability at a site[6,7]. But mtDNA analysis is one of a number of instances (see Discussion) in which scoring allelic variation is more complicated, because there are more than the three discrete genotype states found in nuclear DNA. Instead of having two copies of each autosome (chromosomes 1–22), human cells have 100–10,000 separate copies of mtDNA, and different copies of mtDNA may differ in DNA sequence at any base. Thus, the conventional nuclear DNA variant caller must be adapted to identify mtDNA variants.

We describe an algorithm specifically tailored to identify mtDNA variants from sequencing data, and apply it to 2,077 participants in the SardiNIA project[8]. We analyze both homoplasmies (conventionally defined as variants affecting all of the mtDNA copies within a cell compared to a standard sequence) and heteroplasmies (defined as the presence of a mixture of more than one type of mtDNA within a cell). We examine transition/transversion ratios, coding vs. noncoding changes, and changes with age.

Analyses are extended with a method to assess mtDNA copy number. Copy number is a critical determinant of mitochondrial function and has been proposed as a potential biomarker for disease. For example, studies have shown that elevated mtDNA copy number is associated with cancer risk[9,10]. Given that there are two copies of autosomal DNA in a cell, our method infers mtDNA copy numbers based on the observed ratios of sequence coverages between mtDNA and autosomal DNA. We estimate the heritability of copy number and show its correlations with gender, age, and waist circumference and waist-hip ratio.

## Materials and Methods

### Ethics statement

All participants gave written informed consent, with protocols approved by institutional review board of the National Institute on Aging (04-AG-N317).

### mtDNA variant caller

Recent analyses of mtDNA variants[11,12] have taken an approach that determines homoplasmic and heteroplasmic sites directly based on allele counts of sequence reads. This approach does not account for error rates in sequence reads, and hence potentially results in both false positive and false negative variant calls. We propose a likelihood-based model that takes into account the sequencing error rate at each base in each sequence read. The algorithm builds on the conventional autosomal DNA variant callers[6,7], but is modified to allow for allele fractions (i.e., heteroplasmic levels) at a variant site to vary across individuals.

#### Likelihood-based model.

We aim to predict the genotype at each mtDNA position, one individual at a time; therefore, the subscripts indicating mtDNA position and individual are suppressed for simplicity. At each mtDNA base position, we use ***G*** to denote one individual’s genotype. In theory, ***G*** can take 15 possible values {A, C, G, T, A/C, A/G, A/T, C/G, C/T, G/T, A/C/G, A/C/T, A/G/T, C/G/T, A/C/G/T}. If ***G*** takes one of the first four possible genotypes (with a single allele), it is by definition called a homoplasmy. However, by convention, if the genotype is the same as the standard reference allele, it is not counted as a homoplasmy. The remaining 11 possible genotypes are called heteroplasmies. We estimate the posterior probability of having a certain genotype ***G*** given all the observed sequence reads:

P(G|reads)∝P(reads|G)×P(G) (1)

where *P*(***G***|*reads*) is the posterior probability, *P*(*reads*|***G***) is called “genotype likelihood”, and *P*(***G***) is the prior probability of genotype ***G***, which can be assigned by researchers. We can then assign the genotype with highest posterior probability to the site for the individual. So the key is to estimate 15 genotype likelihoods: *P*(*reads*|***G***).

Assuming the mtDNA site of interest is covered by N sequence reads, we use r_i_ to denote the called base from read i (i = 1, 2, …, N) and e_i_ to denote the corresponding sequencing error rate of that base for read i, which can be estimated from the corresponding Phred-like base quality score Qi ($e_{i}=\;10^{-Q_{i}/10}$).

We assume that each sequence read represents an independent random sampling from the multiple copies of mtDNA in a cell, and therefore a genotype likelihood can be estimated by:

$$P(reads|G)=\prod_{i=1}^{N}\;P(r_{i}|G)$$ (2)

When ***G*** has only one allele (i.e. ***G*** ∈ {*A*, *C*, *G*, *T*}), *P*(*r*_*i*_|***G***) can be directly estimated while considering the base error rate:

$$P\left(r_{i}|G\right)=\{\begin{array}{l} 1-e_{i},\;if\;r_{i}=G\\ \;\frac{e_{i}}{3},\;if\;r_{i}\neq G \end{array}$$

Because a cell has multiple copies of mtDNA and each individual can have different fractions for the alleles, when ***G*** has more than one allele (i.e. ***G*** ∈ {*A*/*C*, *A*/*G*, *A*/*T*, *C*/*G*, *C*/*T*, *G*/*T*, *A*/*C*/*G*, *A*/*C*/*T*, *A*/*G*/*T*, *C*/*G*/*T*, *A*/*C*/*G*/*T*}), we need to introduce allele fractions as unknown parameters of interest to be estimated by maximum likelihood. For example, when ***G*** has two alleles *G*_*1*_ and *G*_*2*_, *G*_1_, *G*_2_ ∈ {*A*, *C*, *G*, *T*}, we denote by *f*_*1*_ and *f*_*2*_ the allele fractions of *G*_*1*_ and *G*_*2*_ for the individual, respectively. Then:

$$P\left(r_{i}|G\right)=\{\begin{array}{l} \left(1-e_{i}\right)\times f_{1}+\frac{e_{i}}{3}\times f_{2},\;if\;r_{i}=G_{1}\\ \left(1-e_{i}\right)\times f_{2}+\frac{e_{i}}{3}\times f_{1},\;if\;r_{i}=G_{2}\\ \frac{e_{i}}{3},\;if\;r_{i}\neq G_{1},r_{i}\neq G_{2} \end{array}$$

Because *f*_*1*_+*f*_*2*_ = 1, we effectively have a single unknown parameter in the above equation. Similarly, we can derive the corresponding formulas when ***G*** has three or four alleles with more allele fractions as unknown parameters (see S1 Text).

We next consider all the reads covering the mtDNA site of interest and update the genotype likelihoods as a function of allele fractions using Eq 2. When ***G*** has more than one allele, we maximize the genotype likelihood functions using allele fractions as parameters (the Simplex method for maximization is implemented in our variant calling algorithm). We then use maximized genotype likelihood to estimate posterior probabilities in Eq 1.

We note that a similar idea was recently proposed by Ye et al.[13] to identify mtDNA heteroplasmies in sequences from 1000 Genomes Project participants. Our publicly available software package was developed independently and further accounts for the circularity of the mtDNA genome (see below) and identifies both homoplasmies and heteroplasmies.

#### Accounting for the circularity of the mtDNA genome

**Fig 1:**
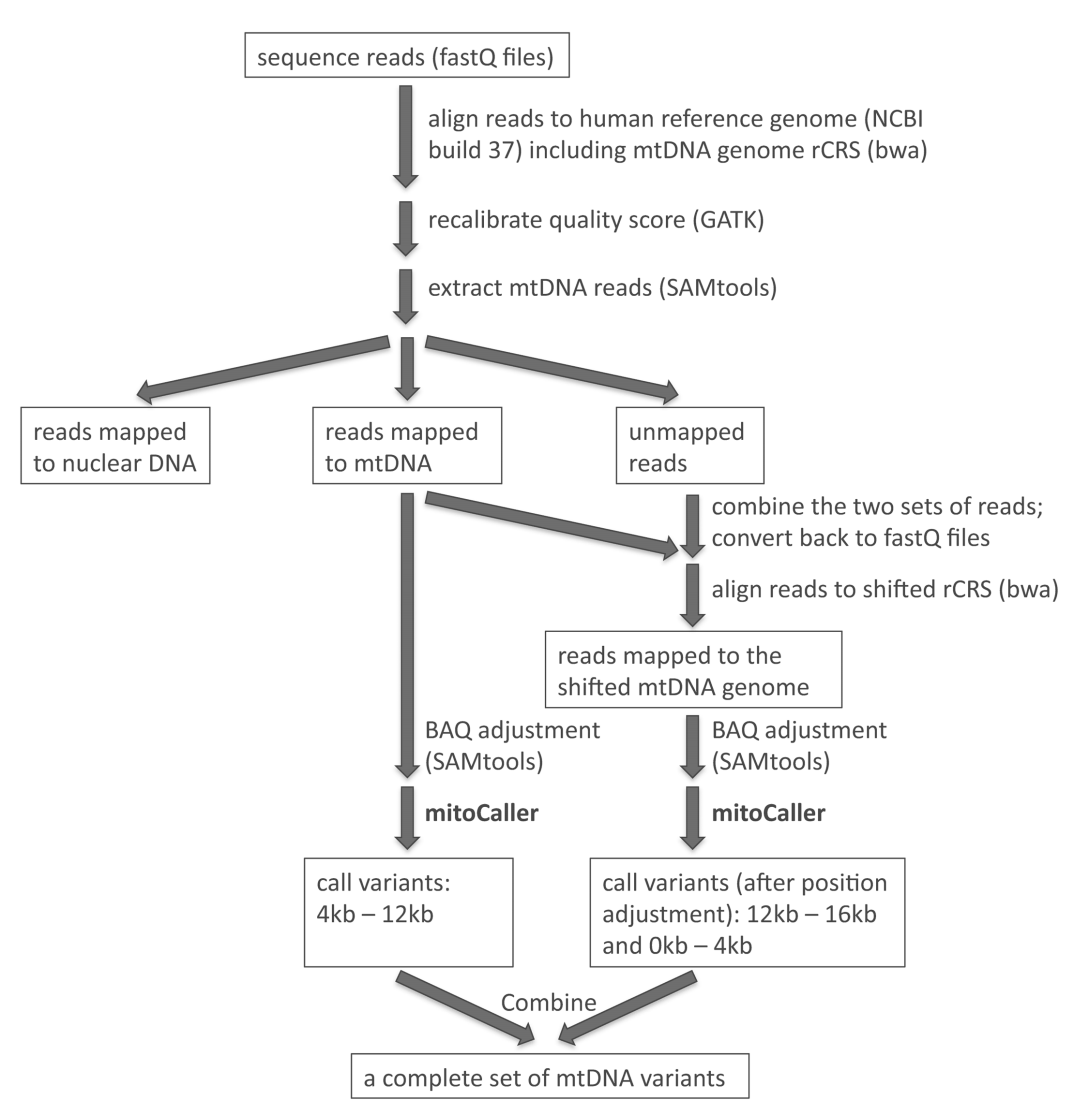
Analysis pipeline to identify mtDNA variants includes aligning sequence reads to the whole genome reference (including mtDNA rCRS reference); extracting mtDNA reads; combining mapped and unmapped reads to do a second alignment to the shifted rCRS reference; applying the mtDNA variant caller separately to the reads mapped to the two linear mtDNA reference genomes; and combining the two sets of called mtDNA variants.

Even though mtDNA has a circular genome, current studies investigating the mtDNA variation from sequencing data employ a linear genome as reference to align sequence reads. Typically, researchers use the revised Cambridge Reference Sequence (rCRS, Gene Bank number NC_012920) as the “mtDNA reference”, with a “breakpoint” introduced in the replication control region to “start” at position 1 and “end” at position 16,569. As a result, sequence reads that link the two “ends” (i.e., those that cover the artificial breakpoint in the circular genome) are not aligned and are discarded. Consequently, the variant caller uses an incomplete set of reads to identify variants. We propose a “double alignment” strategy, using two linear reference genomes in sequence read alignment (S1 Fig). The conventional rCRS is the reference for the first sequence alignment, and variants are then identified only in the internal region of the genome (e.g., from position 4,000 to position 12,000), skipping the two artificial ends of the rCRS. A “shifted” rCRS reference is then created by making a breakpoint in the middle of the circular sequence (position 8,000); the “shifted” rCRS thus starts at the original position 8,000 and ends at the original position 7,999 (S1 Fig). The sequence alignment is then repeated using the shifted rCRS as reference. Intuitively, one could simply align the whole-genome sequence reads to the human nuclear genome plus the shifted rCRS reference, but we propose a more efficient approach that combines the unmapped reads and reads mapped to rCRS reference from the first alignment, and then uses them as the input reads for the second alignment. This approach guarantees that all the reads covering the original break point are included in the analysis. We then use the variant caller to call variants between original positions 1 to 4,000 and between 12,000 to 16,569. Variant calling in these two regions is not affected by the breakpoint at position 8,000. Finally, we combine the two sets of called variants from the two alignments to obtain a complete set of variants for the whole mtDNA genome. The pipeline for the “double alignment” procedure is outlined in Fig 1. When the “double alignment” strategy was applied in our SardiNIA data set, the most significant read depth increase was observed in the 100 bp region on each side of the breakpoint, with a 2.1-fold increase (or raw coverage increase of 114X) in average depth (see S1 Text for more discussion on the coverage).

### Inferring mtDNA variation from whole-genome sequence

The mtDNA variant caller was applied to whole-genome sequence data of 2,077 individuals selected among 6,921 participants in the “SardiNIA” study of the genetics of quantitative traits in the Sardinian founder population [8]. The details on the selection of individuals to be sequenced are in S1 Text.

Sequence data were generated at the University of Michigan Medical School Core Sequencing Lab. DNA was extracted by a standard salting-out method from whole blood samples after a red blood cell lysing step. Libraries were generated from 3–5 μg of genomic DNA using sample preparation kits from Illumina and New England Biolabs. Paired-end sequence reads (typically, 100 to 120 bp in length) were generated with Illumina Genome Analyzer IIx and Illumina HiSeq 2000 instruments. Samples were sequenced to an average depth of 4.2X.

Reads were aligned to the human reference genome (GRCh37 assembly with decoy sequences, as available in the 1000 Genomes Project ftp site, ftp://ftp.1000genomes.ebi.ac.uk) using BWA (version 0.5.9), allowing at most 5 differences (mismatches or gaps), and trimming read tails with average base quality <15. After alignment, base qualities were recalibrated and duplicate reads were flagged and excluded from analysis. We reviewed summary metrics generated using QPLOT and verifyBamId for each aligned sample, to remove samples with low sequencing depth, poor coverage of regions with high or low GC content, or evidence for sample contamination.

We then extracted from all sequence reads those that were uniquely mapped to the mtDNA reference genome by bwa with a mapping quality score ≥20 (i.e., the theoretical probability of wrong alignment ≤1%). The mtDNA variant caller was applied only to these mtDNA reads. Fig 1 outlines the pipeline for aligning sequence reads to the whole genome reference, extracting mtDNA reads, and applying the mtDNA variant caller taking into account the circularity of the mtDNA genome.

In implementing the likelihood-based model to identify mtDNA variants, we also applied quality control filters to help avoid the inclusion of false variants because of sequencing errors. At a position of interest in mtDNA, we considered only reads with base sequencing error rate ≤1% (i.e., recalibrated base quality score ≥20). We also applied sequencing depth filters: we required an overall mtDNA median depth > 100 for an individual to be included in the analysis; and at each base of interest for variant calling, we required a raw depth ≥40 and a depth ≥10 after base quality score filtering at 20. To call a heteroplasmy, we further required that 1) all alleles of the called genotype are observed at least once in both forward and reverse strand sequence reads, and 2) the minor allele fraction (MAF) for an individual is ≥ 4%. The MAF threshold was chosen based on simulations in which we mimicked the SardiNIA sequencing experiments and simulated similar coverage data with reads of comparable quality scores (see S1 Text for details). An MAF threshold of 4% corresponded to an empirical false discovery rate (i.e., the proportion of false heteroplasmies among all identified heteroplasmies) of 2%. We also used data from a deeply sequenced parent-child trio (~80-fold average coverage for nuclear DNA and ~6,000-fold average coverage for mtDNA) to evaluate the accuracy of variant calling for the same three individuals from the low-pass sequencing data. Using the results from deep-sequencing data as gold standards, we confirmed all the heteroplasmies identified in the three individuals by our variant caller with the 4% MAF cut-off (We found no false negatives in the child or the mother of the trio, but did see one false negative in the father). By contrast, if we lowered the MAF cut-off to a less stringent threshold of 1.6%, we observed an average false discovery rate of 30%. Looking at deep sequencing data from a few individuals could not provide a definite guideline about the MAF cut-off, but supports well the choice of a 4% MAF cut-off for this dataset.

We have also considered the possibility that nuclear copies of parts of mtDNA sequence (i.e., nuclear mitochondrial DNA, or NUMTs) might be the source of false positives for heteroplasmies. In our analysis, as mentioned above, we included only reads that were uniquely mapped to mtDNA. With further analyses on sampled individual cases, we found that any representation of NUMTs is minimal, and should therefore not restrict the utility of the method (See S1 Text for a detailed discussion).

We used publicly available online software HaploGrep (http://haplogrep.uibk.ac.at/) to classify SardiNIA individuals into different haplogroups based on mtDNA, and ANNOVAR[14] to annotate the called variants on mtDNA and assess whether homoplasmies and heteroplasmies show different distributions in functional categories.

### Estimating mtDNA copy number from sequencing data

Assuming autosomal and mtDNA are handled and sequenced with no significant differences, average sequencing coverage should be proportional to DNA copy number for autosomal and mtDNA:

$$\frac{mtDNA\;average\;coverage}{mtDNA\;copy\;number\;per\;cell}=\frac{autosomal\;DNA\;average\;coverage}{autosomal\;DNA\;copy\;number\;per\;cell}$$

As a proof of principle, we looked at the average depth of coverage across the 22 autosomal chromosomes for 100 randomly selected individuals and observed that as expected, sequencing depth was largely flat across 22 chromosomes for each individual (S2 Fig).

Because there are two copies of autosomal DNA in a cell, we could infer the mtDNA copy number by:

$$mtDNA\;copy\;number\;per\;cell=\frac{mtDNA\;average\;coverage}{autosomal\;DNA\;average\;coverage}\times 2$$ (3)

We used SAMtools (http://samtools.sourceforge.net/) to obtain the coverage of each base in the genome from the aligned bam[15] files. The average coverages for autosomal DNA and mtDNA were then calculated accordingly.

We applied our computational method to the same whole-genome sequencing data from 2,077 Sardinians and estimated mtDNA copy number for each sample. We also used a NovaQUANT Human Mitochondrial to Nuclear DNA Ratio Kit (EMD Chemicals Inc.) to validate a random group of 18 samples experimentally by qPCR (see S1 Text for more details). We tested for age and gender effects on the mtDNA copy number, and also assessed any association of mtDNA copy number with eleven quantitative traits collected for the cohort that include 5 anthropometric traits (height, weight, BMI, waist circumference, and waist-hip ratio), 2 frailty traits (walking speed and grip strength), and 4 lipid traits (HDL-cholesterol, LDL-cholesterol, total cholesterol, and triglycerides). In addition, POLY software (http://www.sph.umich.edu/csg/chen/public/software/poly/) was used to estimate the heritability of mtDNA copy number based on the known family structure in the SardiNIA cohort.

We note that a similar framework has been suggested by Chu and colleagues[16] to estimate copy number. However, their method identified only reads mapped to mtDNA and counted all the remaining reads as mapped to nuclear DNA. As a result, it would include unmappable reads in calculations and would thus overestimate the nuclear DNA sequence coverage. Furthermore, in calculating the effective length of human genome, their method did not exclude the regions in the nuclear DNA that could not be covered by sequencing. Our computational method avoids those pitfalls and hence should provide more accurate copy number estimates. Here we also validate the method with Q-PCR and implement it to estimate mtDNA copy number in a large-scale population study.

### URLs

Software programs implementing our methods are freely available at http://lgsun.irp.nia.nih.gov/hsgu/software/mitoAnalyzer/index.html.

## Results

### Nature of variants and accumulation of heteroplasmies with age

**Fig 2:**
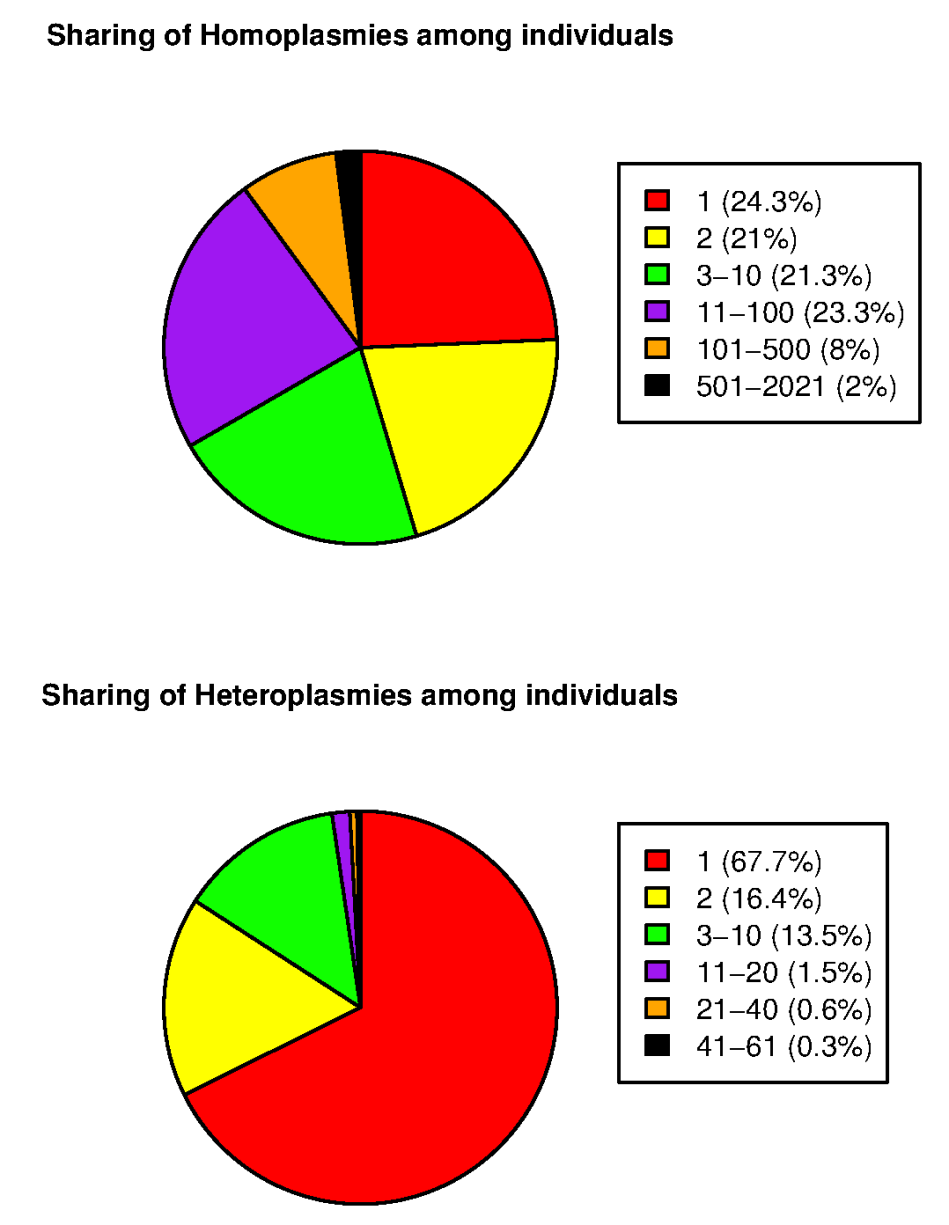
Sharing of mtDNA variants among 2,077 SardiNIA sequencing project participants.

Applying our mtDNA variant calling program to the cohort of 2,077 Sardinians, we identified an overall average of 22.2 homoplasmies and 0.73 heteroplasmies per individual. S1 Table and S2 Table provide complete lists of homoplasmies and heteroplasmies, respectively. S3 Fig shows histograms of the numbers of homoplasmies and heteroplasmies per individual. The distribution of the number of homoplasmies per individual was bimodal. One group showed relatively fewer homoplasmies (mode of 11), whereas the other had a mode of 32. Compared to the current reference phylogenetic tree, the two modes represented different European haplogroups. The former fell into the HV subgroup (the mitochondrial reference genome sequence rCRS also belongs to this subgroup, accounting for the smaller number of homoplasmies) and the latter was predominantly correlated with several other clades, including J, T and K subgroups. Looking at the sharing of the mtDNA variants among the 2,077 individuals, we observed significantly higher sharing of homoplasmies than heteroplasmies (Fig 2). For example, 10% of homoplasmies were shared by more than 100 individuals and 2% of homoplasmies were shared by more than 500. By contrast, only ~1% of heteroplasmies were shared by more than 20 individuals.

**Fig 3:**
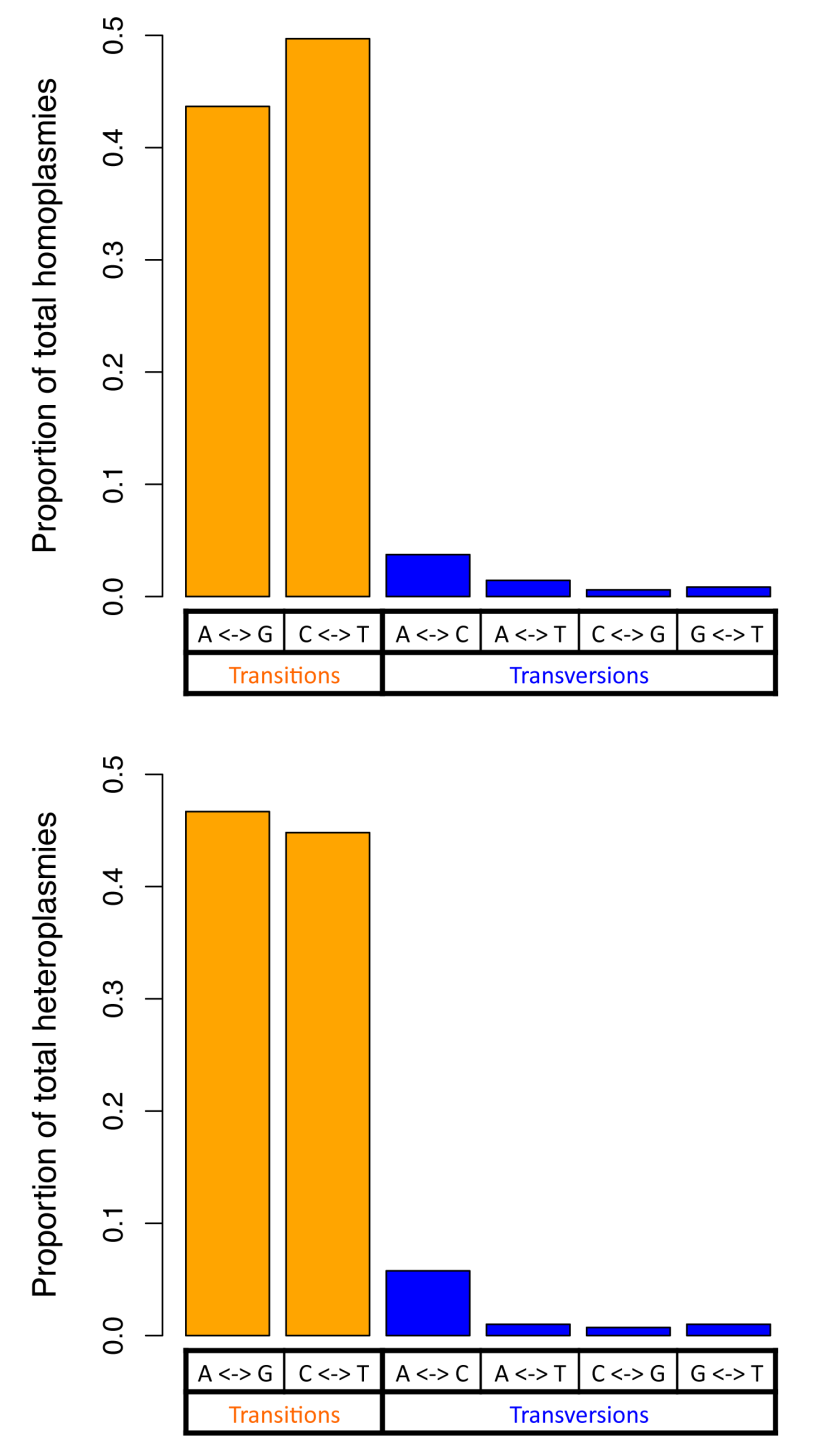
Two classes of base changes for homoplasmies and heteroplasmies.

For both homoplasmies and heteroplasmies, we further investigated numbers of transition and transversion base changes (Fig 3). The transition/transversion ratio was greater than 10 for both homoplasmies and heteroplasmies, which is far higher than the ratio of 2.1 in human nuclear DNA (2.19 in the sequenced Sardinians; see Discussion). Homoplasmies and heteroplasmies showed very similar patterns of base changes (Fig 3; see Discussion).

**Fig 4:**
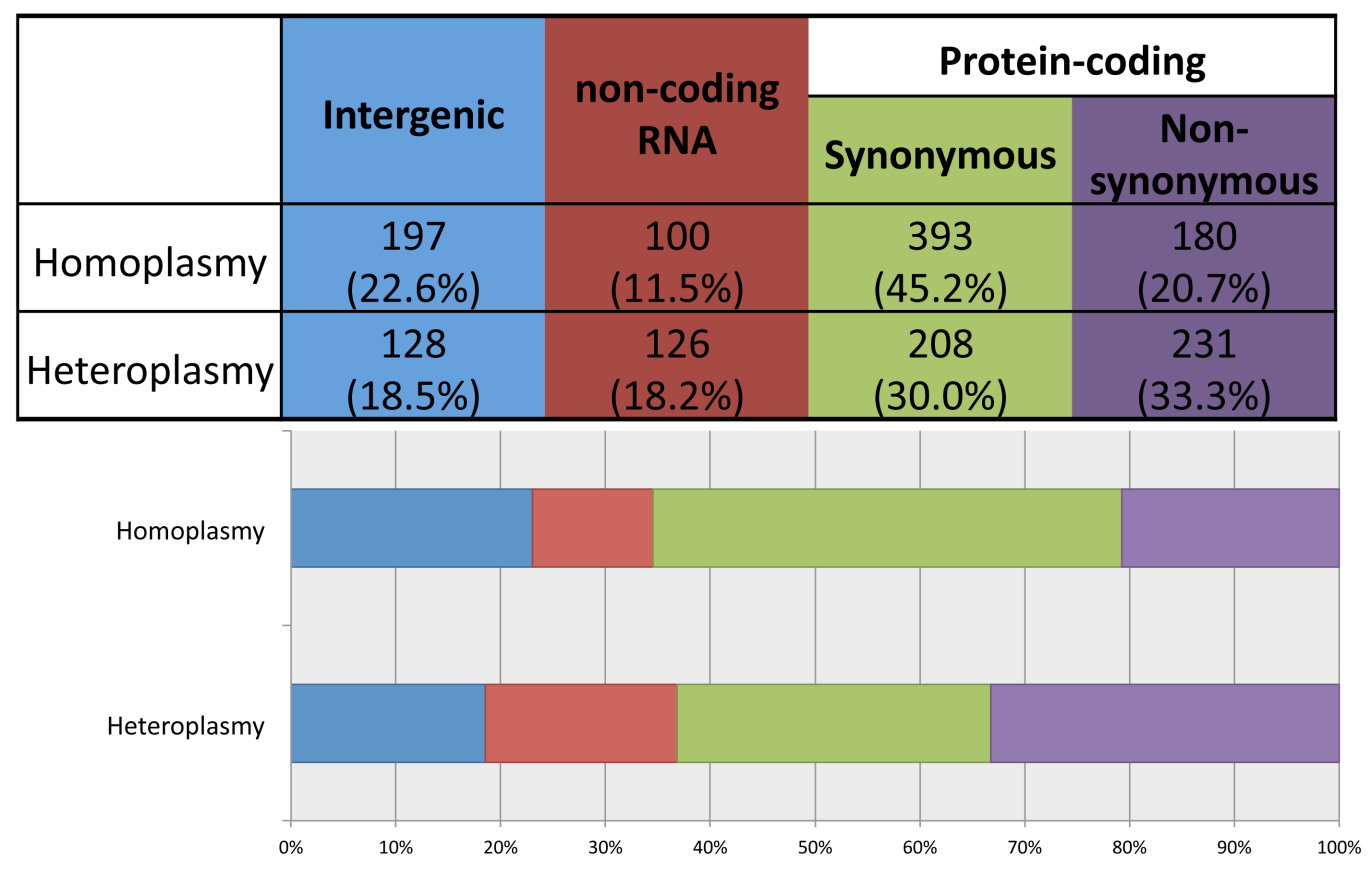
Distribution of homoplasmies and heteroplasmies in four functional categories.

Using ANNOVAR to annotate the identified variants, we grouped them into four functional categories: 1) intergenic; 2) structural RNA(rRNA and tRNA)-encoding; 3) synonymous protein-coding; and 4) non-synonymous protein-coding. As shown in Fig 4, compared to heteroplasmies, homoplasmies are less likely to be RNA-encoding or non-synonymous (chi-square test p-value = 3×10^−13^), consistent with the notion that heteroplasmies represent new mutations and that natural selection makes it challenging for deleterious variants to become fixed as homoplasmies.

**Fig 5:**
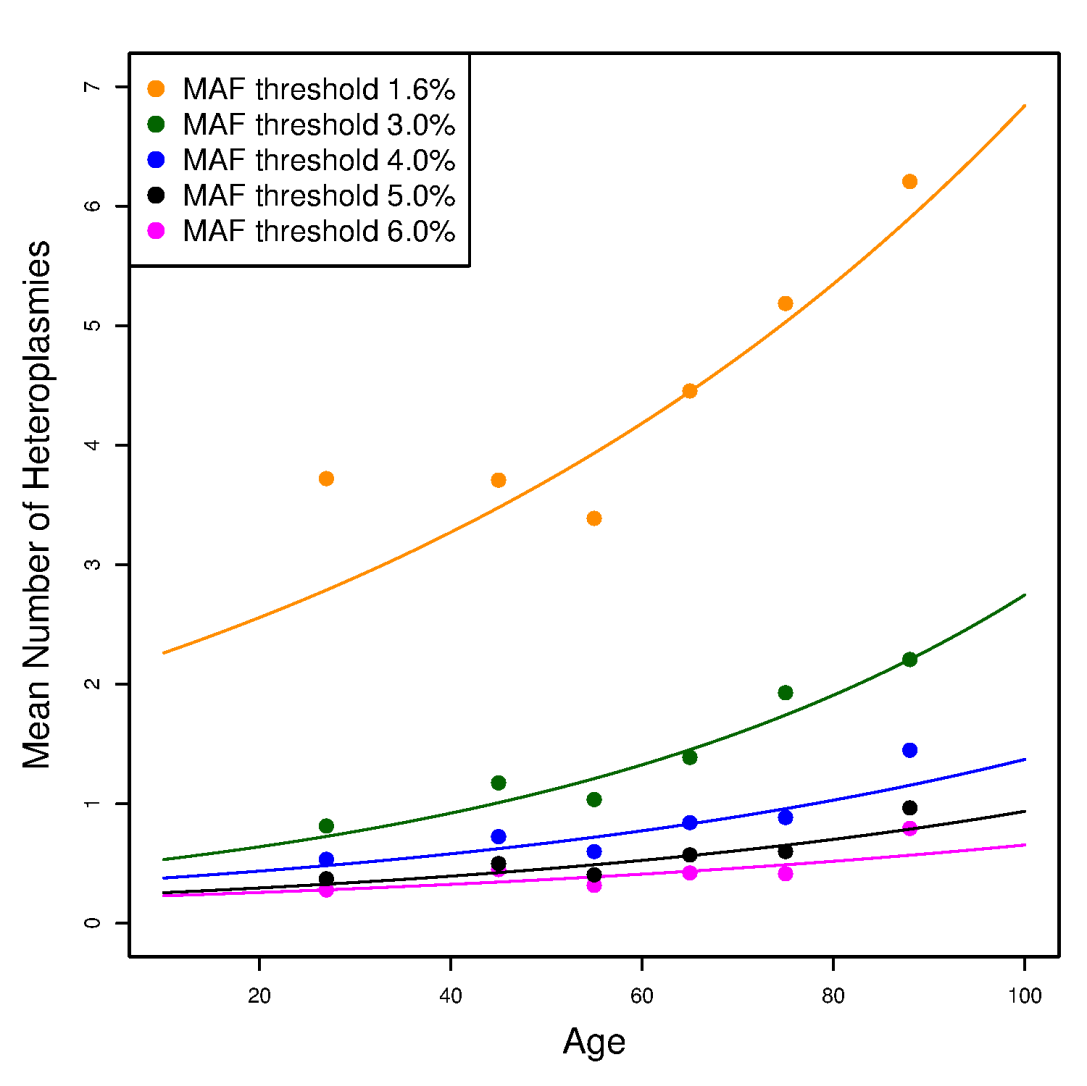
The effect of age on the number of heteroplasmies in the unrelated SardiNIA sequencing project participants. The number of heteroplasmies increases with age with different (colored) minor allele fraction (MAF) thresholds. Each line plots the expected number of heteroplasmies based on the Poisson loglinear model against age at an MAF threshold; while the points represent the observed mean number of heteroplasmies in each age group (<40, 40–50, 50–60, 60–70, 70–80, >80).

To assess any age effect on the number of mtDNA variants, we used a Poisson loglinear model to test the association between the number of variants and age among unrelated individuals (see S1 Text for the procedure to select unrelated individuals from the whole cohort). We observed no relationship between age and the number of homoplasmies (S4 Fig). By contrast, with a minor allele fraction threshold of 4%, we observed a significant increasing trend of the number of heteroplasmies with age (p-value = 6.2×10^−5^). The increasing slope is small, yielding an average increase of ~1 heteroplasmy between ages 20 and 90 (Fig 5), but the slope increased and became more significant when we repeated the analyses with MAF thresholds of 1.6% and 3% (Fig 5; p-values equal to 1.1×10^−15^ and 2.7×10^−11^, respectively). When lowering the MAF threshold, one expects to include more true heteroplasmies together with more false positives. However, false heteroplasmies have no likely relationship with age, whereas the additional true heteroplasmies will strengthen the trend. On the other hand, when we raised the MAF threshold to 5% and 6% (i.e., applying more stringent thresholds), the trend remained but p-values were less significant (8.9×10^−4^ and 0.017, respectively). Thus, given the minor allele fraction thresholds applied, there is appreciable accumulation of heteroplasmies with age (see Discussion).

### Inheritance of mtDNA variants

We used 333 parent-child trios included in the cohort to investigate the sharing of mtDNA variants between parents and their children and to assess features of the inheritance pattern of mtDNA variants. Children and their mothers share essentially all homoplasmies. We observed 7,273 homoplasmic sites in 333 children, among which 7,238 (99.5%) were also observed in their mothers (Table 1). At the same time, 2,940 (40.4%) homoplasmies were also observed in their fathers. This observation is not incompatible with maternal inheritance, because many homoplasmies are shared across the Sardinian population, and thus children and their fathers could share many by chance. This is further supported by the observation that fathers also shared ~40% of mothers’ homoplasmies (Table 1). Concerning heteroplasmies, we observed 207 heteroplasmic sites in 333 children, among which 66 (31.9%) were observed in their mothers and 1 (0.4%) was observed in their fathers. These results indicate that children inherit a proportion of heteroplasmies from their mothers, whereas new heteroplasmies arise both in their own and in their mothers’ lymphocytes during life. The sharing of a single heteroplasmy between a child and its father could well have occurred by chance or may represent rare true patrilineal transmission. Further discussion on the inheritance of heteroplasmies can be found in S1 Text.

**Table 1:** The sharing of mtDNA variants in parent-child trios.

		Comparing child with parents			Comparing mother with father	
	# families	# variants in Child	# shared with mother	# shared with father	# variants in mother	# shared with father
Homoplasmies	333	7273	7238 (99.5%)	2940 (40.4%)	7266	2937 (40.4%)
Heteroplasmies	333	207	66 (31.9%)	1 (0.4%)	206	0 (0%)

### Estimates of mtDNA copy number

Estimating mtDNA copy number for each sample, we observed a range from 50 to 350, with most individuals between 75 and 150 (with mean of 111.5 and standard deviation of 25.0; histogram in S5 Fig). To assess copy number by a standard biochemical assay for comparison, we carried out Q-PCR experimental validation for 18 randomly chosen samples. The Q-PCR measures have considerable intrinsic variability of their own; but taking an average of two experiments, the computational estimates and experimental measurements shared a similar range (scatterplot in S6 Fig), with a correlation of 0.82.

### Correlations of copy number with gender, age, and waist and waist-hip ratio

We observed a significant gender effect on mtDNA copy number: on average, females have 6.7 (6.2%) more copies of mtDNA than males (p-value = 1.6×10^−9^). After adjusting for gender and average sequencing coverage, the estimated mtDNA copy number decreases significantly with age (p-value = 2.7×10^−6^, with an expected 1.5 copy number decrease for every 10 years of age increase, S7 Fig).

When testing the association of mtDNA copy number with a set of 11 quantitative traits, after adjusting for age, gender, and average sequencing coverage, we observed that mtDNA copy number is significantly associated with waist circumference (p-value = 0.0031; after adjusting for multiple tests, Bonferroni-corrected p-value = 0.034) and waist-hip ratio (p-value = 2.4×10^−5^; Bonferroni-corrected p-value = 2.6×10^−4^), but not with BMI (p-value = 0.42) or two frailty-related traits (walking speed, p-value = 0.32; grip strength, p-value = 0.11). We also estimated the heritability of mtDNA copy number as 54%, implying strong genetic regulation of mtDNA level.

## Discussion

### mtDNA sequence variation

The method presented here improves mtDNA variant calling in two ways. First, it directly models sequencing error rates (i.e., uncertainty) in likelihood calculations, and therefore more accurately identifies mtDNA variants and estimates minor allele fractions of heteroplasmies in individuals. Second, the pipeline is adapted to the circular mtDNA genome, aligning sequencing reads to two linear mtDNA reference genomes by a “double-alignment” strategy. This strategy greatly increases the coverage in the hypervariable “junction” region—very important, for example, for phylogenetic studies—that otherwise would have very poor coverage, and can also be useful in aligning sequence reads of other circular genomes.

Recently, several pioneering studies have also used next-generation sequencing technologies to assess mtDNA heteroplasmies. These include several studies applying deep sequencing to amplified mtDNA [11,12,17,18] and others performing whole-genome sequencing and then extracting mtDNA sequence reads [4,13]. Most of these studies used a set of filters to account for sequencing errors and technical artifacts in heteroplasmy identification, which generally included number of mismatches, mapping quality, base quality, minimum depth, double strand validation, and minor allele fraction (MAF). S3 Table compares criteria for calling heteroplasmy among these studies and ours.

Not surprisingly, depending on the sequencing coverage, different studies applied different MAF thresholds in calling heteroplasmies, which makes a direct comparison of results impossible. To do a fair comparison, we applied to five studies a common MAF threshold of 10%—the maximum threshold used in any of the studies. In our study, at least one heteroplasmy is observed in 21.8% of individuals. This is very comparable to the value of 24.4% found by Li and colleagues [12] in 133 individuals sequenced with a mean coverage of 85X, and to 23.1% observed by Rebolledo-Jaramillo and colleagues [18] after sequencing amplified mtDNA from 39 mother-child pairs to 20,000-fold coverage with additional steps to exclude PCR and sequencing errors. In addition, their high-quality data showed that on average one person possessed about 1 heteroplasmy with MAF > 1%, which is in good agreement with our findings of an average of 0.73 heteroplasmies per person with MAF > 4% based on direct sequencing of total DNA.

By contrast, we note that the 1000 Genomes Pilot Project sequenced 163 individuals and found that 45% possessed heteroplasmies with an MAF cut-off at 10% [4]. Similarly, Ye et al. [13] found the prevalence of heteroplasmy to be 44.4% in the full 1,085 member cohort of 1000 Genomes Project. And a sixth study sequenced 40 HapMap individuals and found 65% possessing heteroplasmies with an MAF cut-off at 9% [19]. As a possible explanation of the difference in heteroplasmy prevalence between the two sets of studies, it is suggestive that the first three studies (including ours) used DNA samples extracted directly from cells or tissues, whereas the second set of three studies used DNA samples from transformed (lymphoblastoid) cell lines. Heteroplasmies could thus arise during the expansion of cell lines in culture; and if so, it may be prudent to assess mtDNA content level and heteroplasmies from untransformed cells.

The extent of recovery of alleles in the Sardinian cohort at ~180-fold average coverage (see below) is robust enough to infer some other salient characteristics of mtDNA variation. The analyses indicate that the inheritance pattern of mtDNA variants can be explained by maternal inheritance of homoplasmies and a portion of heteroplasmies, with further heteroplasmic sites arising during life in both children and mothers up to the time of cross-sectional sampling of the cohort. This is consistent with observations from deep sequencing data for two CEPH families[11]. By looking at 333 trios, our study showed that 31.9% of heteroplasmies in children were inherited from their mothers, consistent with the study of Rebolledo-Jaramillo et al. [18], who found an inherited proportion of heteroplasmies (with MAF > 1%) of 28.9% and 27.7% in blood and buccal cells, respectively (results inferred from their Supplementary Materials).

Considering the nature of the allelic variants, the transition/transversion ratios for homoplasmies and heteroplasmies are both about 10, about 5-fold higher than the ratio observed in human nuclear DNA (~2.1). This is consistent with the transition/transversion ratio estimated for mitochondrial mutations occurring at a frequency ≤ 1%[20], and is also consistent with the hypothesis that misincorporation by DNA polymerase gamma and the deamination of nucleotides are major sources of the base changes[20]. Homoplasmies and heteroplasmies share similar distributions among the different types of base change (Fig 3), indicating that the mechanism creating base changes is likely to be the same for homoplasmies and heteroplasmies. In addition, compared to homoplasmies, heteroplasmies are enriched at RNA encoding and non-synonymous protein-coding sites, consistent with new mutations being more likely to be detrimental than those that survive selection during fixation.

As for the much-discussed possibility that heteroplasmies may accumulate with age, we did observe a significant trend. To the best of our knowledge, this is the first direct analysis relating mtDNA variant number with age in a large-scale population study [We note that Rebolledo-Jaramillo et al.[18] compared 39 mother-child pairs and show a similar positive association in mothers but not in children, perhaps because the children were too young or the sample size was too small]. However, estimation of the true rate of increase requires further identification of lower-level heteroplasmies. As mentioned above, to recognize true heteroplasmies against a background of sequencing errors, we applied a conservative minor allele fraction threshold in calling heteroplasmic sites and required that the minor and major alleles are observed from both forward and reverse strands. These filters were necessitated by the level of average mtDNA coverage in each sample. The filters could be relaxed in several ways–for example, by increasing read coverage by deep sequencing of total DNA. As another example, Rebolledo-Jaramillo et al.[18] lowered the heteroplasmy threshold to 1% by analyzing mtDNA purified and amplified before sequencing. That approach, however, requires additional experimental procedures and eliminates the possibility of the simultaneous determination of mtDNA copy number. Additional approaches could use family structures to expand available reads or sequence DNA from single cells, which would assess only 100–1,000 mtDNA molecules, instead of that number augmented by millions of copies from all the cells in a lymphocyte sample. The algorithm could extend the power of analysis using other study designs as well. In particular, longitudinal analysis of a study cohort would be informative, but would require repeated sampling over time.

Several approaches could also improve the variant calling algorithm itself. Calling variants by considering multiple individuals jointly or by considering linkage disequilibrium (LD) structure among variants would probably add little to variant calling accuracy, because we observed that heteroplasmies are rarely shared among individuals (Fig 2). But extensions that detect insertions and deletions (i.e., indels, etc.) as well as single base variants could provide a more complete catalogue of variation.

### mtDNA copy numbers and their dynamics

In assessing mtDNA copy number based on the sequencing coverage ratio between mtDNA and autosomal DNA, we assume that no significant differences are generated between reads of mtDNA and autosomal DNA during the sequencing and processing steps that include genomic DNA fragmentation, adapter ligation, PCR amplification, sequencing, and sequence alignment/mapping. The relatively high correlation with Q-PCR measurements supports this assumption, and the relatively high heritability of copy number (54%) is also in accord with the reliability of the estimates.

We were thus encouraged to use inferred mtDNA copy number in further analyses. We observed that females on average have slightly but significantly higher mtDNA copy numbers than males (6.7 more copies), and the average mtDNA copy number decreases with age, consistent with general decline of cellular energy metabolism during aging and with observations of other researchers in independent cohorts [21]. We note that lymphocytes, the cells studied here, have characteristically low levels of cytoplasm, and the average number of mtDNA molecules per cell is relatively low (average of 111.5) compared to what may be expected in actively growing cells. In fact, further applications of our method to data from the 1000 Genomes Project, which sequenced DNA from transformed lymphocytes, observed an average number of mtDNA copies that is ~6-fold greater (work in progress). This is consistent with the increased mtDNA copy number associated with augmented risk of several types of cancer[9,10].

The correlations of mtDNA copy number with waist circumference and waist-hip ratio, but not with BMI (nor in further assessments, with height or lipid levels; Table 2), are intriguing. The findings hint that mtDNA copy number may also be relatively associated with central obesity and body fat distribution, though further study is needed to find the basis for the correlation.

The relatively high heritability of copy number (54%) is also suggestive. Future analyses, including genome-wide association studies, should identify genetic factors underlying this substantial heritability.

**Table 2:** The effect of mtDNA copy number on a set of anthropometric, frailty, and lipid traits.

Traits	Testing the effect of copy number		
	Effect*	t value	p-value
Height	0.0025	0.44	0.66
Weight	-0.0059	-0.55	0.58
BMI	-0.0032	-0.80	0.42
Waist	-0.0296	-2.96	0.0031
Waist-hip ratio	-0.00028	-4.24	0.000024
Walking speed	-0.00022	-1.00	0.32
Grip strength	0.0130	1.60	0.11
HDL-cholesterol	-0.0099	-0.75	0.46
LDL-cholesterol	-0.0082	-0.25	0.80

*Effect is the slope of the linear regression model, which corresponds to the expected mean change of the trait per one unit increase of the mtDNA copy number. The regression models also include age, sex, and mean nuclear DNA sequence coverage as covariates.

### Future applications

Although our variant calling algorithm was designed to identify mtDNA variants from sequencing data, its potential utility can easily extend to other instances of comparable sequence heterogeneity. For example, such allelic heterogeneity is a major characteristic of cancer, where mutations are expected to occur in only a subset of the sequenced cells because of tumor heterogeneity. Our method could be implemented to detect somatic mutations in cancer cells with allele fractions ranging between 0 and 1 (a similar framework has recently been proposed[22]). As another example, although the extent of RNA editing is unclear[23–27], the method has the potential to study RNA editing from sequencing data and infer the editing level—another parameter that ranges between 0 and 1. The method could also be adapted to the analysis of cell-free fetal DNA in maternal plasma (i.e. a non-invasive way to sequence human fetus[28]), where an important step is to estimate the proportion that is fetal in origin in DNA isolated from maternal plasma during pregnancy.

Overall, our study with ~2,000 individuals is the largest population-scale study of mtDNA variation thus far, and the application of the two mtDNA analysis tools to the SardiNIA study indicates that when whole-genome sequencing data are available, a set of analyses on mtDNA variants and copy numbers can be performed with no additional experimental cost. Many population studies collecting large-scale sequencing data could thus extend the analyses of genetic factors affecting mtDNA levels, their inheritance, and their relation to aging and disease.

## Supporting Information

S1 Fig The “double alignment” strategy using two linear reference genomes to account for the circularity of the mtDNA genome in sequence read alignments. Variants are called in the blue region of each linear reference genome.
(PDF)

S2 Fig Average sequencing coverages across 22 autosomal chromosomes for 100 randomly selected Sardinians. (PDF)

S3 Fig Distributions of the numbers of homoplasmies and heteroplasmies per individual. (PDF)

S4 Fig The effect of age on the number of homoplasmies in SardiNIA sequencing project participants (based on a Poission regression model). (PDF)

S5 Fig Distribution of the estimated mtDNA copy numbers in ~2,000 Sardinians. (PDF)

S6 Fig Scatterplot of computational and experimental estimates of mtDNA copy numbers for 18 randomly selected samples (correlation coefficient: 0.82). (PDF)

S7 Fig The estimated mtDNA copy numbers decrease with age. (PDF)

S8 Fig Sequencing depth across the mtDNA genome for 10 randomly selected individuals. (PDF)

S9 Fig The scatterplot of alternative allele fractions (AAF) of inherited heteroplasmies in children and their mothers. (PDF)

S10 Fig Histograms of MAFs in children for inherited and non-inherited heteroplasmies. (PDF)

S1 Table A list of homoplasmies identified in the Sardinian cohort. (XLSX)

S2 Table A list of heteroplasmies identified in the Sardinian cohort. (XLSX)

S3 Table Comparison of criteria for calling heteroplasmy among multiple studies. (XLSX)

S4 Table A list of heteroplasmies shared by children and their mothers in 333 trios. (XLSX)

S5 Table Results from mapping reads supporting heteroplasmies to NUMTs. (DOCX)

S1 Text Supplementary materials and methods. (DOCX)
