# Correction: Assessing Mitochondrial DNA Variation and Copy Number in Lymphocytes of ~2,000 Sardinians Using Tailored Sequencing Analysis Tools

**DOI:** 10.1371/journal.pgen.1005549

**Published:** 2015-09-29

**Authors:** Jun Ding, Carlo Sidore, Thomas J. Butler, Mary Kate Wing, Yong Qian, Osorio Meirelles, Fabio Busonero, Lam C. Tsoi, Andrea Maschio, Andrea Angius, Hyun Min Kang, Ramaiah Nagaraja, Francesco Cucca, Gonçalo R. Abecasis, David Schlessinger

S1 Table, as originally submitted, incorrectly omits the frequencies of mtDNA homoplasmies and includes data not related to the publication. The correct S1 Table can be viewed below, which provides the required frequencies of each variant for this study.

## Supporting Information

S1 Table A list of homoplasmies identified in the Sardinian cohort. (XLSX)
