# Supplementary material for: Assessing Mitochondrial DNA Variation and Copy Number in Lymphocytes of ~2,000 Sardinians Using Tailored Sequencing Analysis Tools
Source: PLoS Genet. 2015 Jul 14;11(7):e1005306. doi: 10.1371/journal.pgen.1005306 (PMC4501845; doi:10.1371/journal.pgen.1005306)
Supplement: S1 Fig — Variants are called in the blue region of each linear reference genome. (PDF) [file pgen.1005306.s001.pdf]

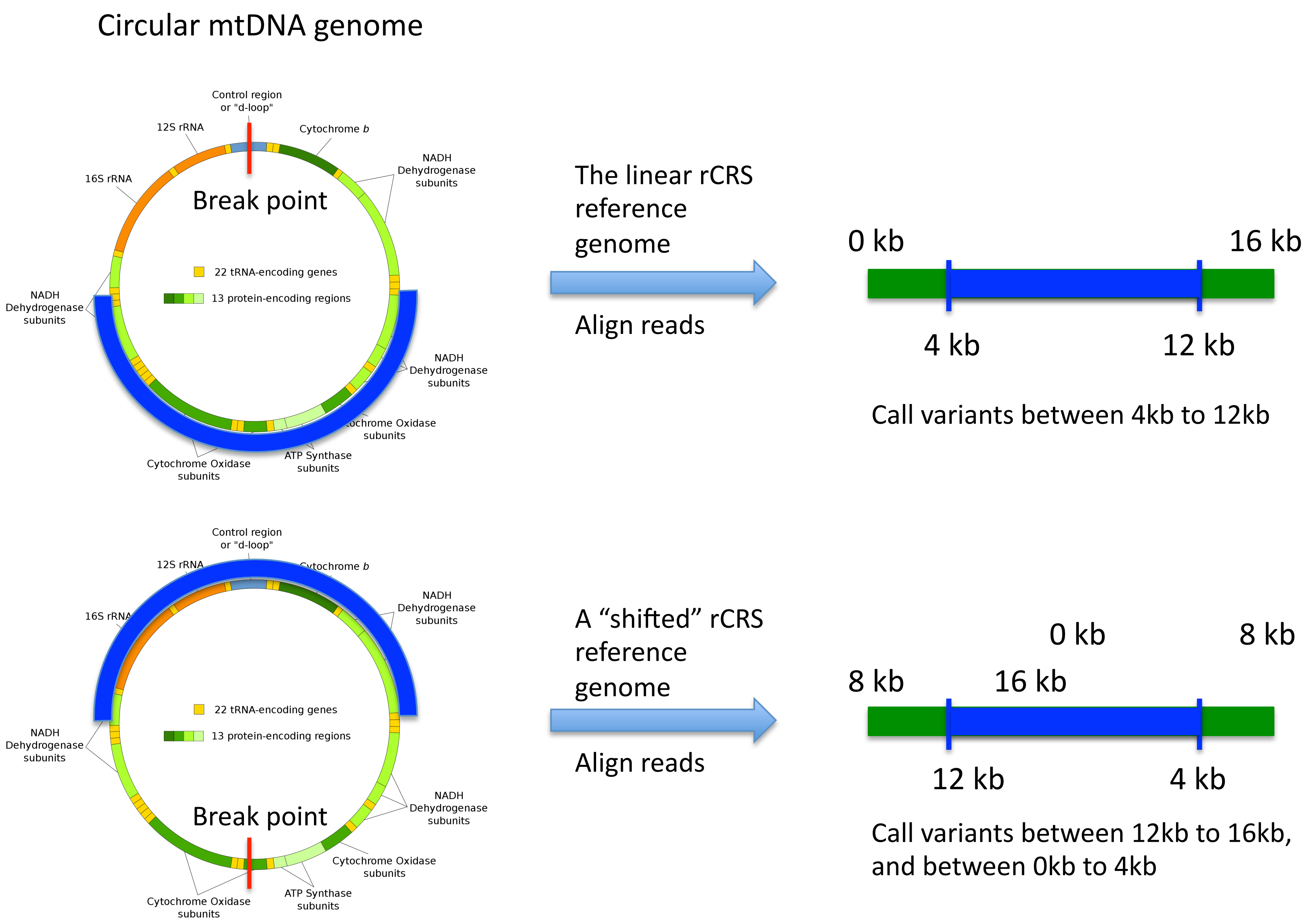
Circular mtDNA genome
Control region or "d-loop"
12S rRNA
Cytochrome b
16S rRNA
NADH Dehydrogenase subunits
Break point
22 tRNA-encoding genes
13 protein-encoding regions
Cytochrome Oxidase subunits
ATP Synthase subunits
The linear rCRS reference genome
Align reads
0 kb
16 kb
4 kb
12 kb
Call variants between 4kb to 12kb
A “shifted” rCRS reference genome
Align reads
0 kb
8 kb
8 kb
16 kb
12 kb
4 kb
Call variants between 12kb to 16kb, and between 0kb to 4kb
