# Supplementary figures and images for: Assessing Mitochondrial DNA Variation and Copy Number in Lymphocytes of ~2,000 Sardinians Using Tailored Sequencing Analysis Tools
Source: PLoS Genet. 2015 Jul 14;11(7):e1005306. doi: 10.1371/journal.pgen.1005306 (PMC4501845; doi:10.1371/journal.pgen.1005306)

**Coverage of 22 chromosomes for 100 samples**

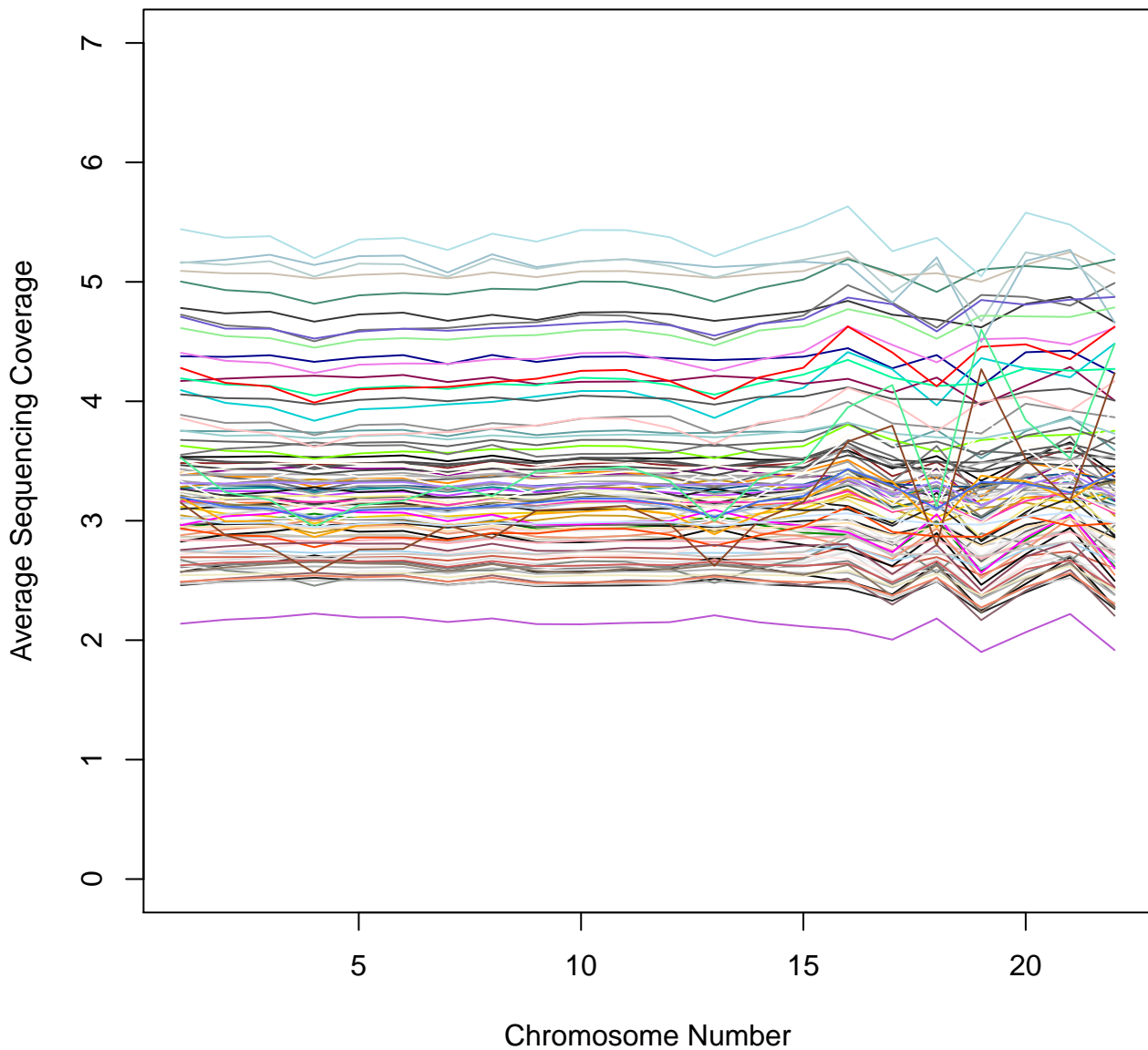

Supplement: S2 Fig — (PDF) [file pgen.1005306.s002.pdf]

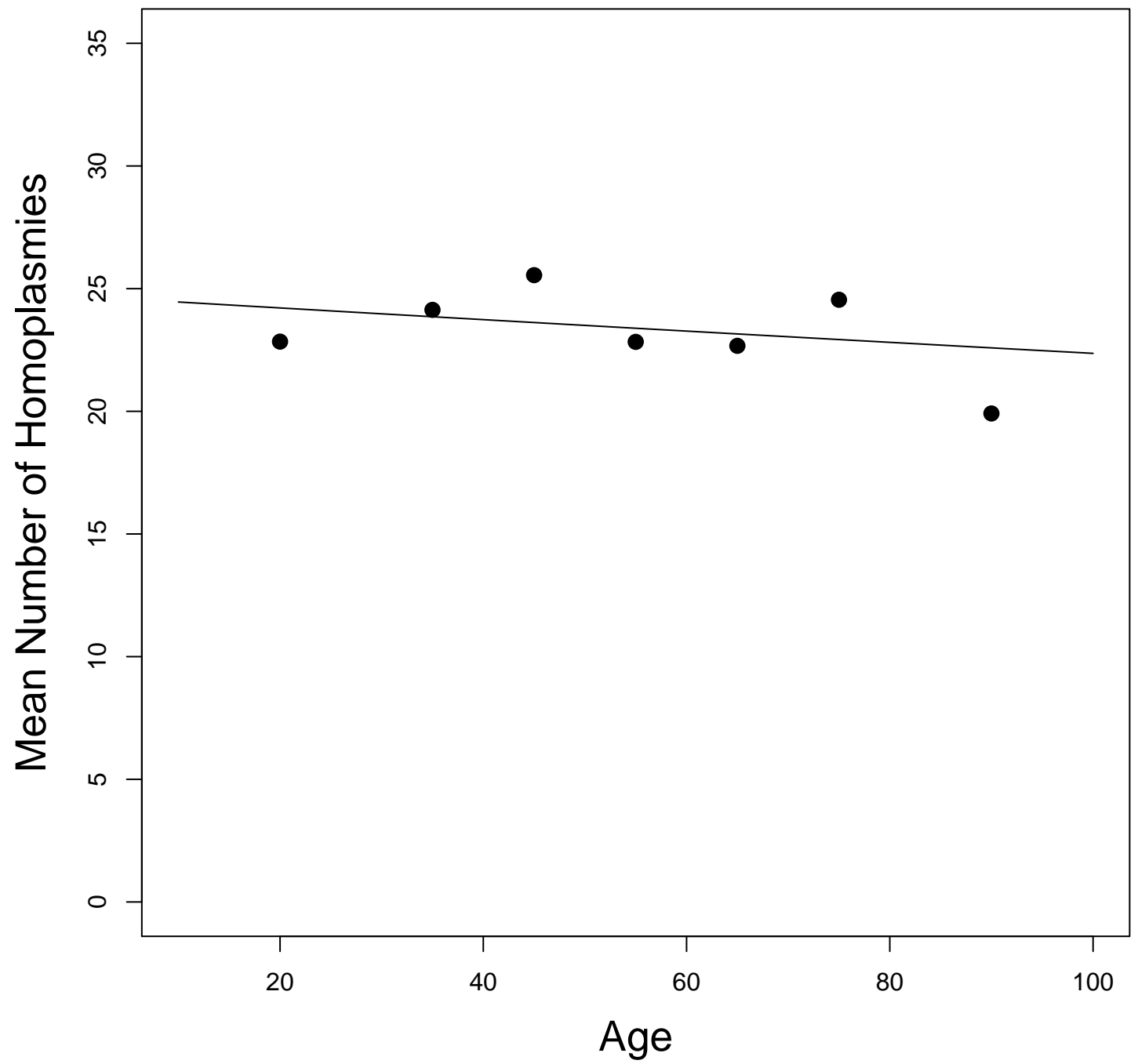

Supplement: S4 Fig — (PDF) [file pgen.1005306.s004.pdf]

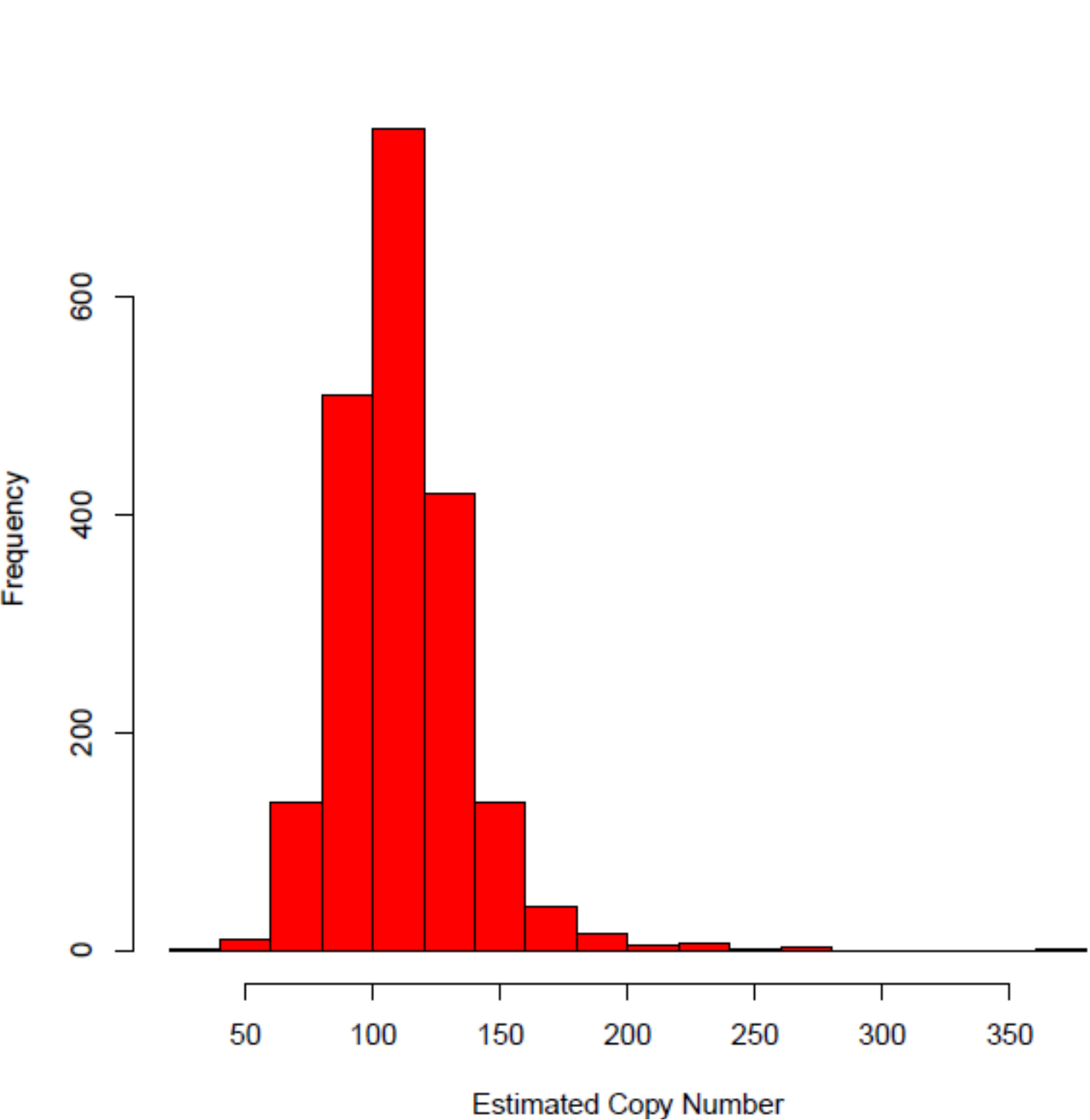

Supplement: S5 Fig — (PDF) [file pgen.1005306.s005.pdf]

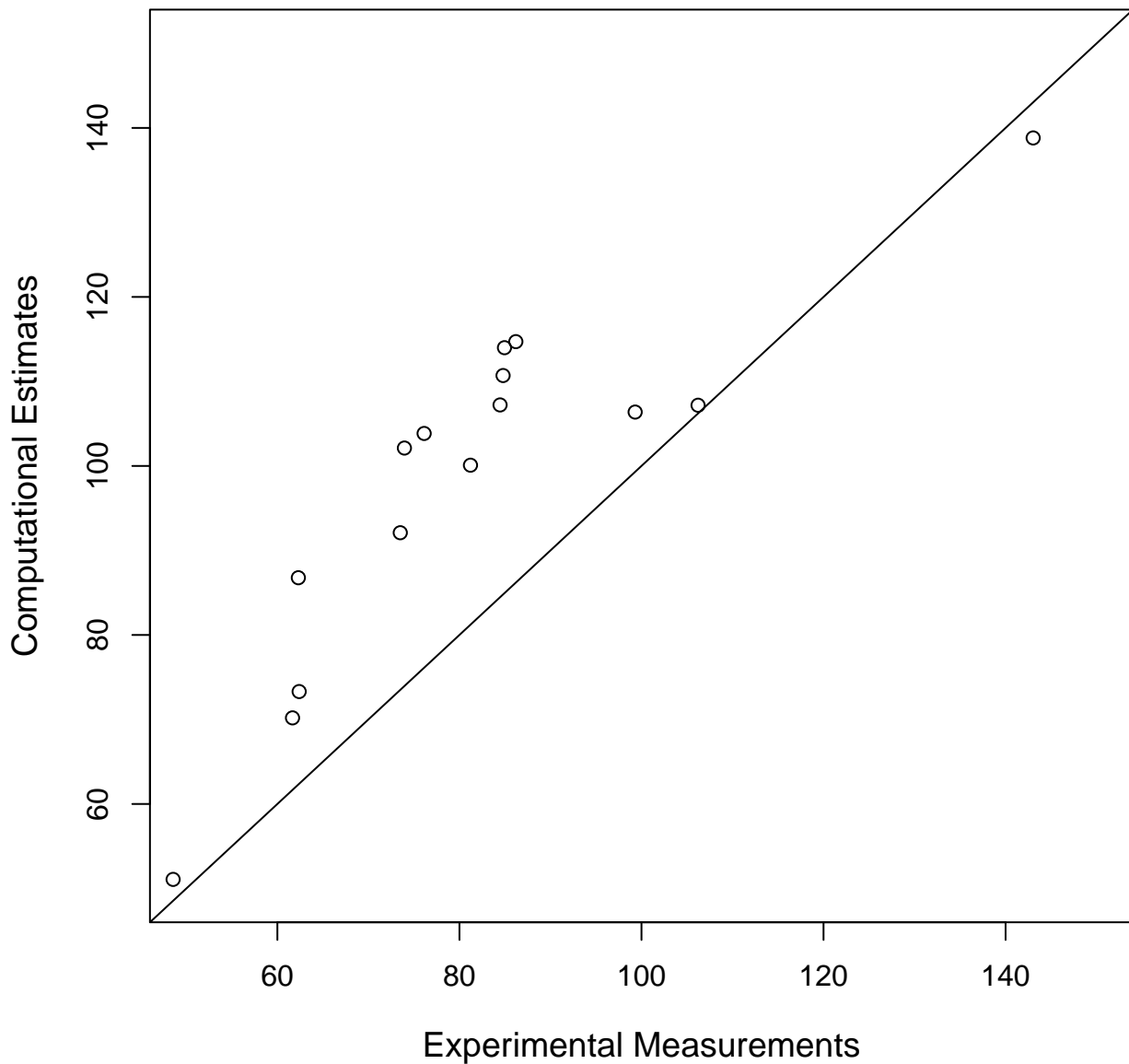

Supplement: S6 Fig — (PDF) [file pgen.1005306.s006.pdf]

**Age versus Estimated Copy Number of 2000 Samples**

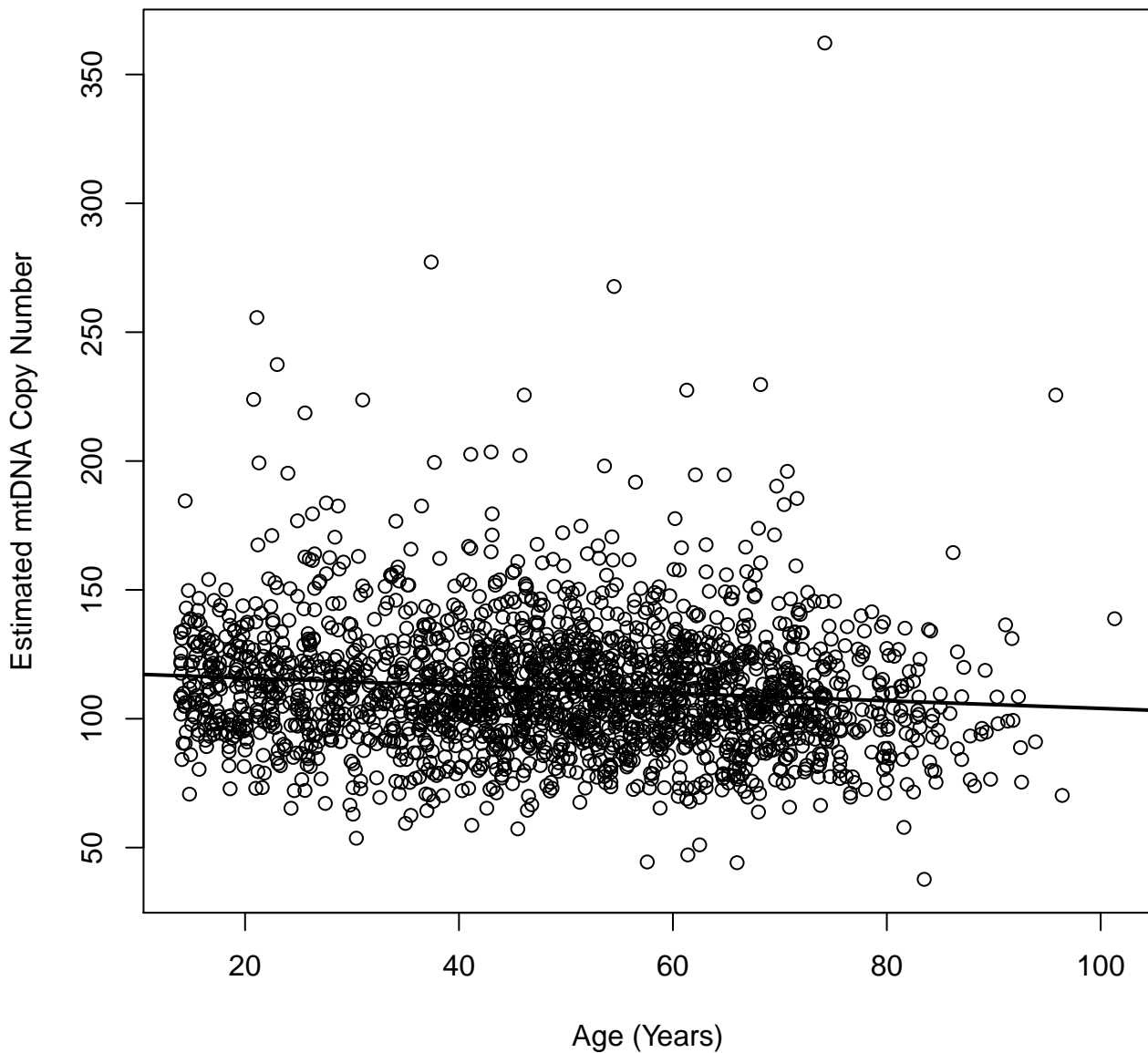

Supplement: S7 Fig — (PDF) [file pgen.1005306.s007.pdf]

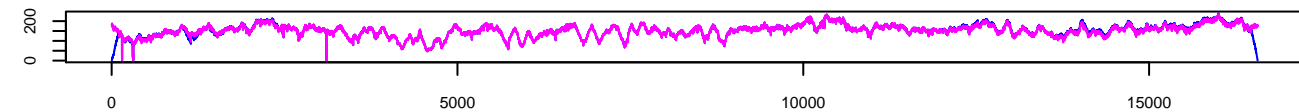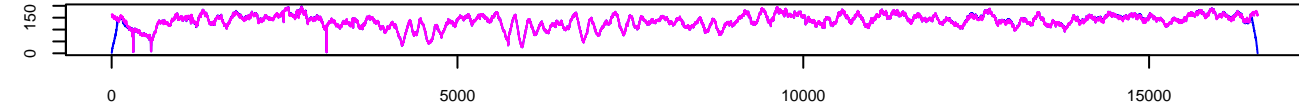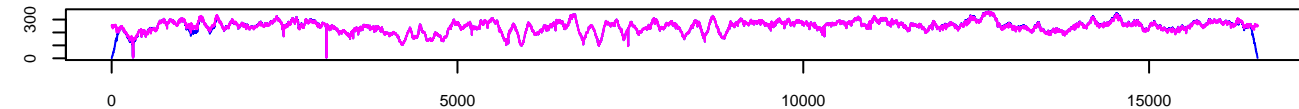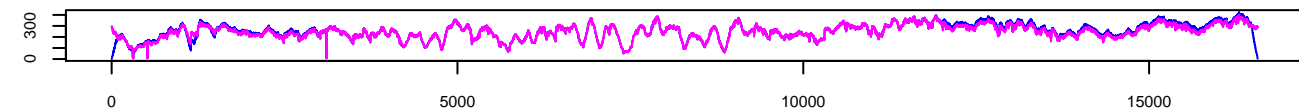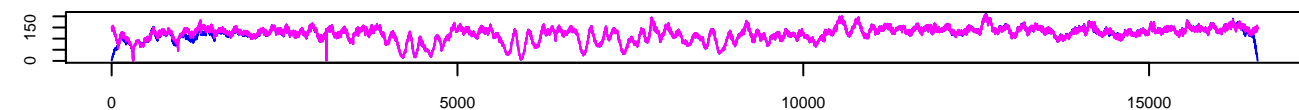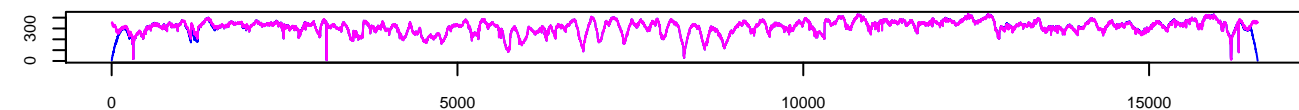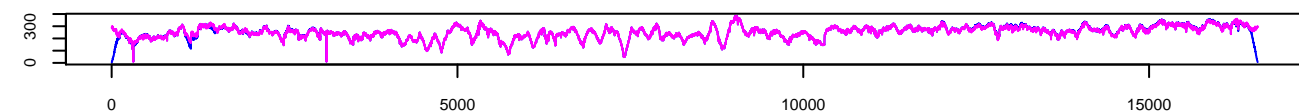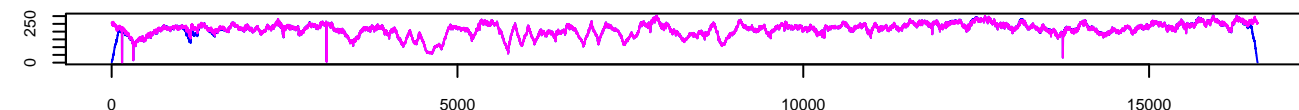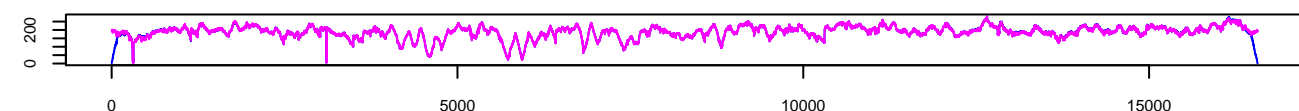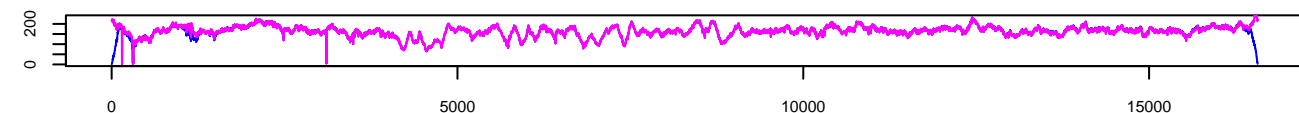

Supplement: S8 Fig — (PDF) [file pgen.1005306.s008.pdf]

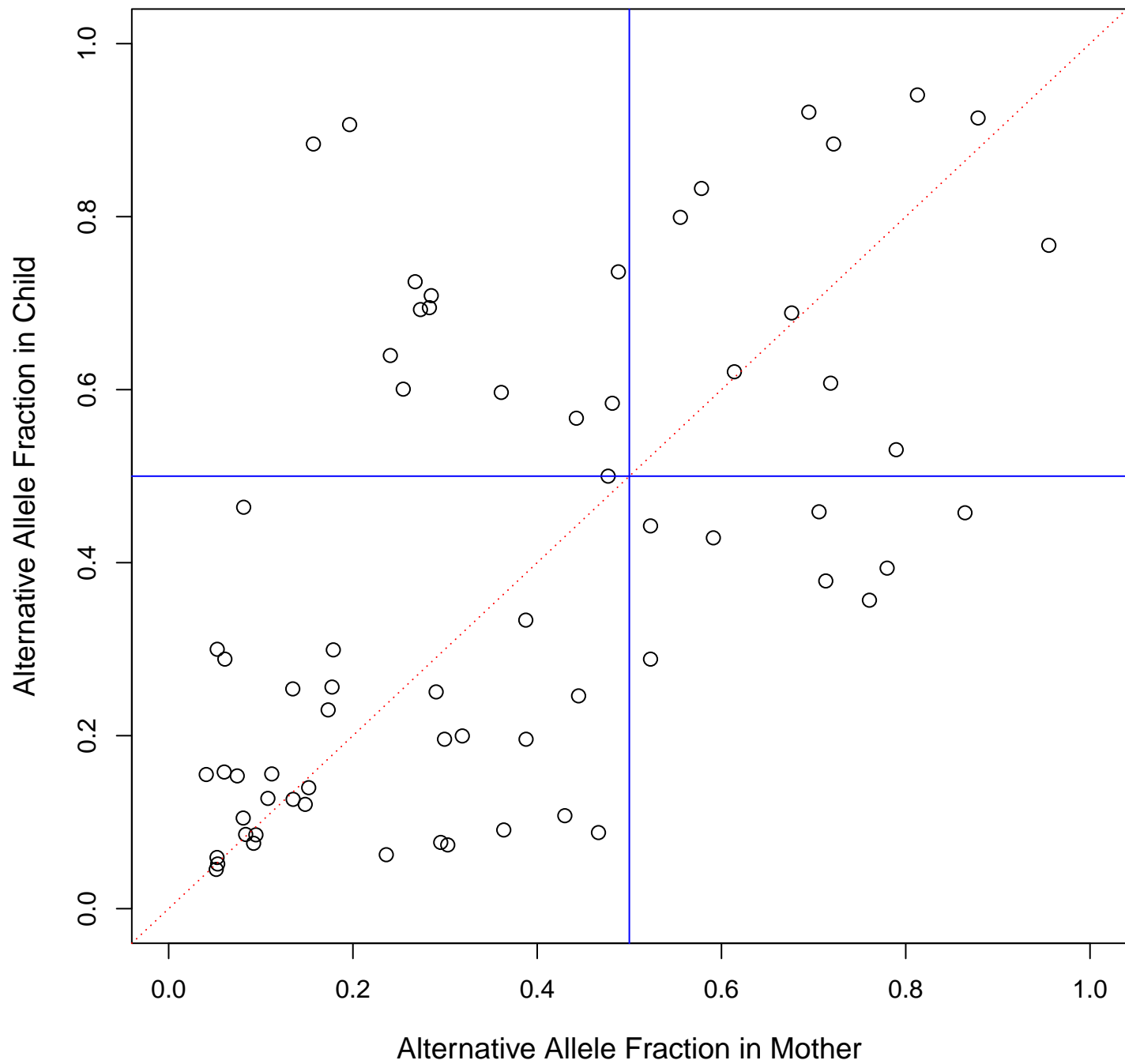

Supplement: S9 Fig — (PDF) [file pgen.1005306.s009.pdf]

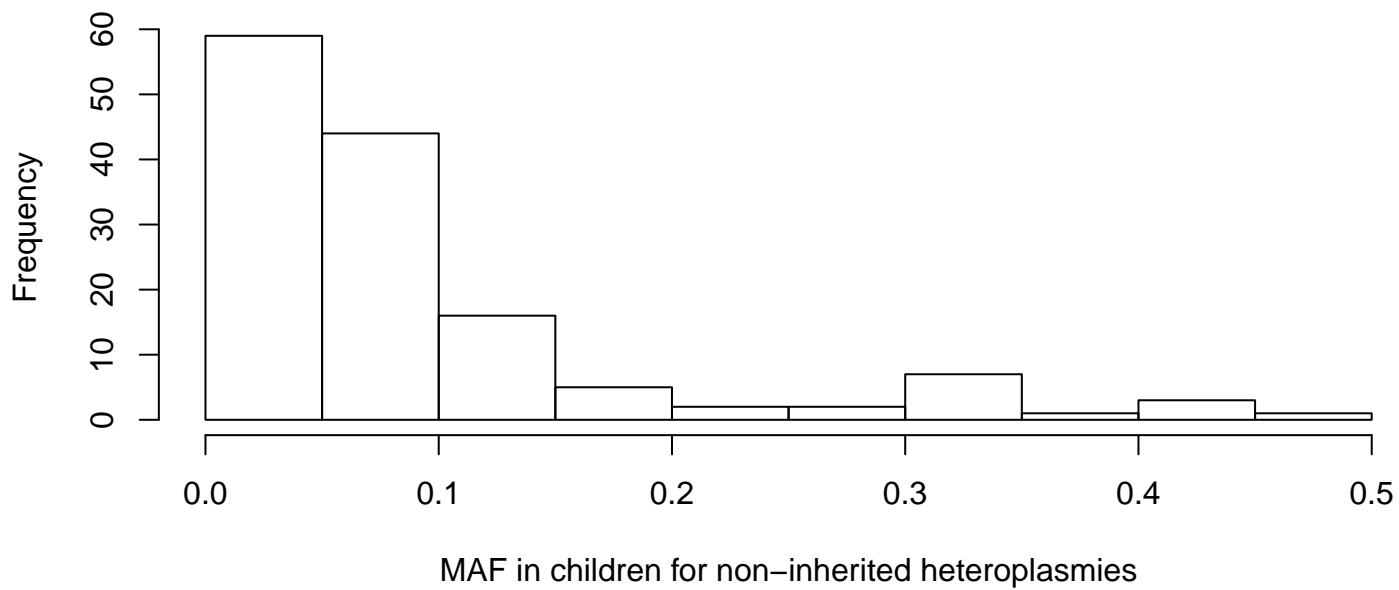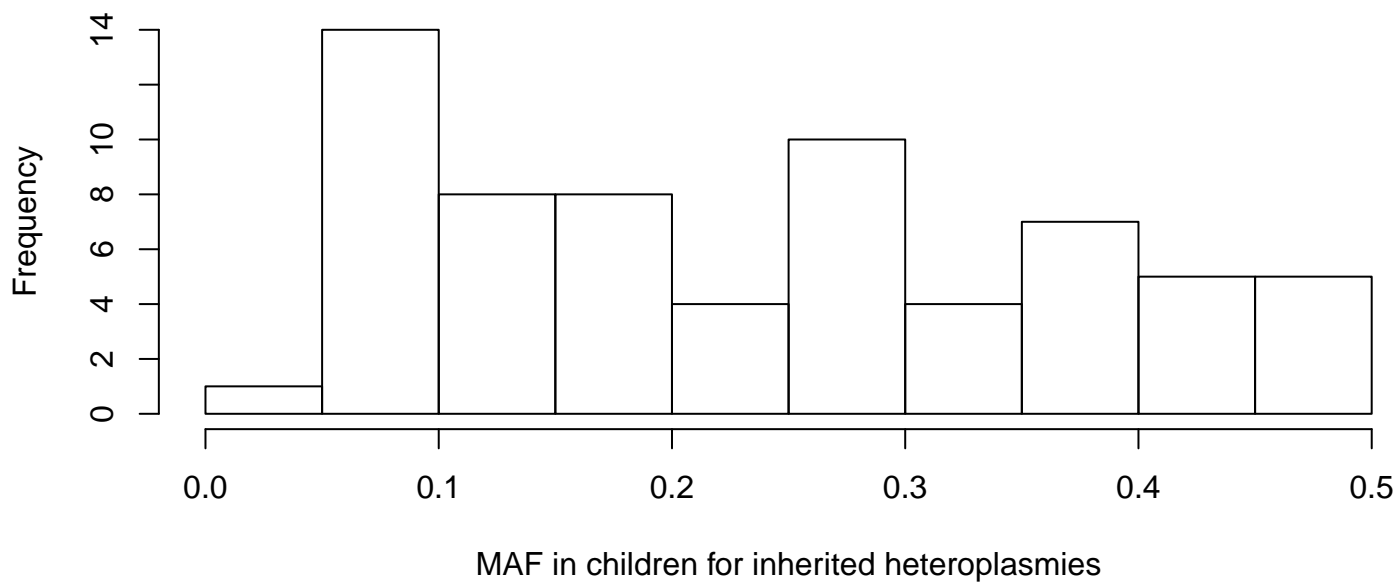

Supplement: S10 Fig — (PDF) [file pgen.1005306.s010.pdf]
