# Supplementary material for: Assessing Mitochondrial DNA Variation and Copy Number in Lymphocytes of ~2,000 Sardinians Using Tailored Sequencing Analysis Tools
Source: PLoS Genet. 2015 Jul 14;11(7):e1005306. doi: 10.1371/journal.pgen.1005306 (PMC4501845; doi:10.1371/journal.pgen.1005306)
Supplement: S3 Fig — (PDF) [file pgen.1005306.s003.pdf]

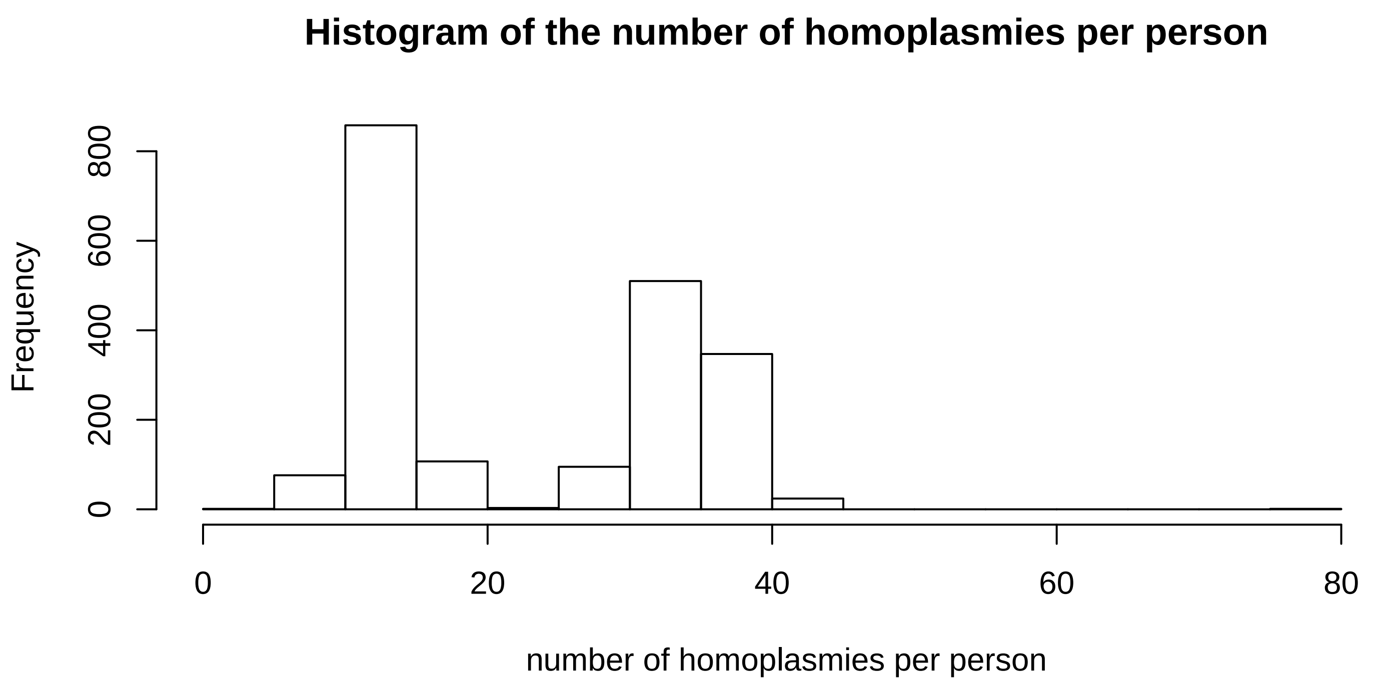

Histogram of the number of homoplasmies per person
Frequency
0
200
400
600
800
0
20
40
60
80
number of homoplasmies per person

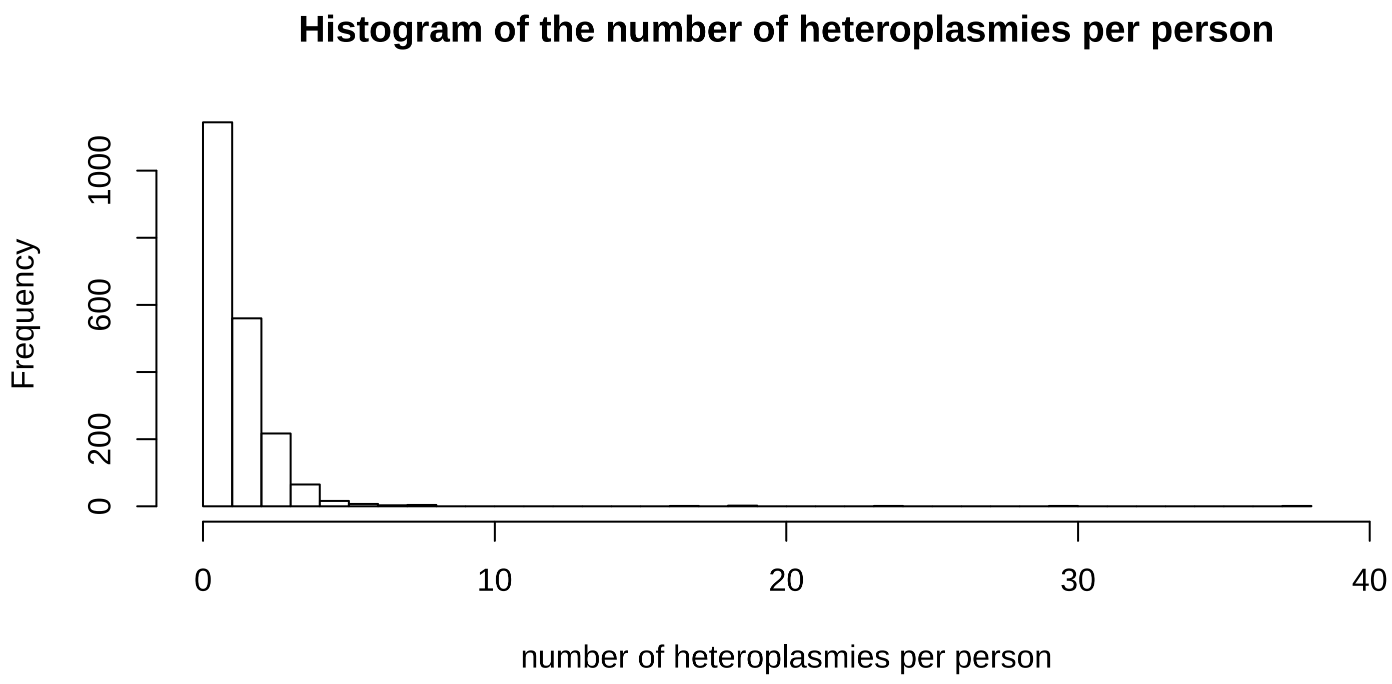

Histogram of the number of heteroplasmies per person
Frequency
0
200
600
1000
0
10
20
30
40
number of heteroplasmies per person
