# Supplementary material for: Assessing Mitochondrial DNA Variation and Copy Number in Lymphocytes of ~2,000 Sardinians Using Tailored Sequencing Analysis Tools
Source: PLoS Genet. 2015 Jul 14;11(7):e1005306. doi: 10.1371/journal.pgen.1005306 (PMC4501845; doi:10.1371/journal.pgen.1005306)
Supplement: S5 Table — (DOCX) [file pgen.1005306.s015.docx]

**Supplementary Table S5**. Results from mapping reads supporting heteroplasmies to NUMTs

| Subject ID | Pos | REF | Genotype Call | Allele Fractions | Reads (QS>20) supporting the **ALT** allele | | | | Reads (QS>20) supporting the **REF** allele | | | |
| --- | --- | --- | --- | --- | --- | --- | --- | --- | --- | --- | --- | --- |
|  |  |  |  |  | # reads | Mapping results | | | # reads | Mapping results | | |
|  |  |  |  |  |  | Not mappable or EMM* >=3 | EMM=2 | EMM=1 |  | Not mappable or EMM >=3 | EMM=2 | EMM=1 |
| 12006 | 295 | C | C/T | 0.051/0.949 | 93 | 93 | 0 | 0 | 5 | 5 | 0 | 0 |
| 12006 | 10398 | A | A/G | 0.040/0.960 | 189 | 189 | 0 | 0 | 8 | 8 | 0 | 0 |
| 160 | 6227 | T | C/T | 0.204/0.796 | 25 | 23 | 2 | 0 | 97 | 81 | 10 | 6 |
| 21025 | 95 | A | C/T | 0.220/0.770 | 44 (C) | 44 | 0 | 0 | 153 (T) | 153 | 0 | 0 |
| 2444 | 4949 | A | A/G | 0.943/0.057 | 19 | 18 | 1 | 0 | 314 | 293 | 20 | 1 |
| 2444 | 14818 | A | A/G | 0.335/0.665 | 155 | 155 | 0 | 0 | 78 | 78 | 0 | 0 |
| 28200 | 961 | T | C/T | 0.399/0.601 | 18 | 18 | 0 | 0 | 27 | 27 | 0 | 0 |
| 28200 | 3492 | A | A/C | 0.922/0.078 | 6 | 6 | 0 | 0 | 69 | 69 | 0 | 0 |
| 3160 | 16183 | A | A/C | 0.645/0.355 | 5 | 5 | 0 | 0 | 9 | 9 | 0 | 0 |
| 3160 | 16296 | C | C/T | 0.075/0.925 | 74 | 74 | 0 | 0 | 6 | 6 | 0 | 0 |
| 36460 | 6750 | C | C/T | 0.386/0.614 | 129 | 52 | 19 | 58 | 81 | 43 | 12 | 26 |
| 36460 | 8265 | T | C/T | 0.054/0.946 | 9 | 4 | 3 | 2 | 154 | 74 | 40 | 40 |
| 39318 | 1673 | T | C/T | 0.278/0.722 | 59 | 53 | 4 | 2 | 153 | 153 | 0 | 0 |
| 42971 | 2735 | G | A/G | 0.134/0.860 | 23 | 23 | 0 | 0 | 141 | 141 | 0 | 0 |
| 42971 | 3489 | A | A/G | 0.956/0.044 | 6 | 6 | 0 | 0 | 127 | 127 | 0 | 0 |
| 42971 | 7079 | C | C/T | 0.954/0.046 | 8 | 2 | 6 | 0 | 162 | 50 | 106 | 6 |
| 42971 | 10245 | T | C/T | 0.156/0.844 | 24 | 24 | 0 | 0 | 129 | 129 | 0 | 0 |
| 42971 | 15562 | A | A/G | 0.842/0.158 | 32 | 32 | 0 | 0 | 170 | 170 | 0 | 0 |
| 5655 | 8838 | G | A/G | 0.297/0.703 | 33 | 7 | 3 | 23 | 78 | 15 | 21 | 42 |
|  | | | | |  |  |  |  |  |  |  |  |
| **Total** (combining 19 sites) | | | | | 951 | 828 | 38 | 85 | 1961 | 1631 | 209 | 121 |
| Percentage | | | | | 100% | 87.1% | 4.0% | 8.9% | 100% | 83.2% | 10.7% | 6.2% |
| **Grand Total** (combining **ALT** + **REF** reads at 19 sites) | | | | | 2912 | 2459 | 247 | 206 |  |  |  |  |
| Percentage | | | | | 100% | 84.4% | 8.5% | 7.1% |  |  |  |  |

NOTE: * EMM (**E**xtra **M**is-**M**atches) = #mismatches for best alignment to NUMTs - #mismatches for alignment to mtDNA for a pair of reads
