# Supplementary material for: Assessing Mitochondrial DNA Variation and Copy Number in Lymphocytes of ~2,000 Sardinians Using Tailored Sequencing Analysis Tools
Source: PLoS Genet. 2015 Jul 14;11(7):e1005306. doi: 10.1371/journal.pgen.1005306 (PMC4501845; doi:10.1371/journal.pgen.1005306)
Supplement: S1 Text — (DOCX) [file pgen.1005306.s016.docx]

**S1 Text: Supplementary Materials and Methods**

**1.** **The formula for genotype likelihood estimation when the underlying genotype *G* has three or four alleles**

When ***G*** has three alleles *G_1_*, *G_2_*, and *G_3_*, $G_{1},G_{2},G_{3}\in\{A,C,G,T\}$, we denote by *f_1_*, *f_2_*, and *f_3_* the allele fractions of *G_1_*, *G_2_*, and *G_3_* for the individual, respectively. *f_1_*+*f_2_*+*f_3_* =1. Then:

$$P\left( r_{i} | \boldsymbol{G} \right)=\left\{ \begin{aligned} \left( 1-e_{i} \right)\times f_{1}+\frac{e_{i}}{3}\times f_{2}+\frac{e_{i}}{3}\times f_{3}, if r_{i}=G_{1} \\ \left( 1-e_{i} \right)\times f_{2}+\frac{e_{i}}{3}\times f_{1}+\frac{e_{i}}{3}\times f_{3}, if r_{i}=G_{2} \\ \left( 1-e_{i} \right)\times f_{3}+\frac{e_{i}}{3}\times f_{1}+\frac{e_{i}}{3}\times f_{2}, if r_{i}=G_{3} \\ \frac{e_{i}}{3}, if r_{i}\neq G_{1}{,r}_{i}\neq G_{2}{,r}_{i}\neq G_{3} \end{aligned} \right.$$

When ***G*** has four alleles *G_1_*, *G_2_*, *G_3_*, and *G_4_*, $G_{1},G_{2},G_{3},G_{4}\in\{A,C,G,T\}$, we denote by *f_1_*, *f_2_*, *f_3_*, and *f_4_* the allele fractions of *G_1_*, *G_2_*, *G_3_*, and *G_4_* for the individual, respectively. *f_1_*+*f_2_*+*f_3_*+*f_4_*=1. Then:

$$P\left( r_{i} | \boldsymbol{G} \right)=\left\{ \begin{aligned} \left( 1-e_{i} \right)\times f_{1}+\frac{e_{i}}{3}\times f_{2}+\frac{e_{i}}{3}\times f_{3}+\frac{e_{i}}{3}\times f_{4}, if r_{i}=G_{1} \\ \left( 1-e_{i} \right)\times f_{2}+\frac{e_{i}}{3}\times f_{1}+\frac{e_{i}}{3}\times f_{3}+\frac{e_{i}}{3}\times f_{4}, if r_{i}=G_{2} \\ \left( 1-e_{i} \right)\times f_{3}+\frac{e_{i}}{3}\times f_{1}+\frac{e_{i}}{3}\times f_{2}+\frac{e_{i}}{3}\times f_{4}, if r_{i}=G_{3} \\ \left( 1-e_{i} \right)\times f_{4}+\frac{e_{i}}{3}\times f_{1}+\frac{e_{i}}{3}\times f_{2}+\frac{e_{i}}{3}\times f_{3}, if r_{i}=G_{4} \end{aligned} \right.$$

**2. Base-level coverage across mtDNA before and after "double-alignment"**

Supplementary Figure S8 shows the coverage across the mitochondrial genome for a random set of 10 individuals. The blue line represents the coverage with “single alignment” using a linear mtDNA genome; the purple line represents the coverage with “double alignment” taking into account the circular mtDNA genome. The two lines are largely overlapping across the genome except for the first and last 100 base pairs of the mtDNA genome, where the “double alignment” coverage is much higher than the “single alignment”, with a 2.1-fold increase (or raw coverage increase of 114X) in average depth. Based on the plots, the coverage across mtDNA genome is not uniform (because of the complexity of some regions), consistent with the variable coverage for nuclear DNA in whole-genome and exome sequencing.

**3. Details on the selection of individuals to be sequenced in the SardiNIA sequencing project**

Samples to be sequenced were selected in trios, taking advantage of their highly informative content for haplotype reconstruction. Trios (or parent–offspring pairs for incomplete trios) were selected starting from the founders of all available families to assure the representation of all haplotypes that have been propagated within families (Pistis et al. 2014). The selection procedure was performed using ExomePicks (http://genome.sph.umich.edu/wiki/ExomePicks).

**4.**  **Use simulation to determine the minor allele fraction (MAF) threshold for calling heteroplasmies**

We simulated sequence reads such that the sequencing coverage was 180X and per base Phred-like quality scores were uniformly distributed between 20 and 30 (these parameters were chosen to mimic closely the SardiNIA sequencing experiments). In our simulation, each site had only one allele (i.e. no heteroplasmies at all) and heteroplasmies could only be detected because of sequencing errors. We used the procedure of our variant caller to call variants and estimated the expected number of falsely identified heteroplasmies per mtDNA genome (16,569 bp) at different MAF thresholds with 10,000 simulations for each threshold. The following table presents the results for three different MAF thresholds. Considering we were studying ~2,000 mtDNA genomes, the MAF threshold of 4.0% is appropriate. We also estimated based on the table that the MAF threshold of 4.0% corresponded to an empirical false discovery rate (i.e. the proportion of false heteroplasmies among all identified heteroplasmies) of 2%.

| MAF Threshold | 3.0% | 4.0% | 5.0% |
| --- | --- | --- | --- |
| Expected # of errors per mtDNA genome | 1.73 | 0.016 | 0.0009 |

**5. Discussion on the impact of NUMTs (nuclear mitochondrial DNA) on heteroplasmy identification and mtDNA copy number estimation**

To avoid analyzing reads from NUMTs, we included in the analysis only the reads that were uniquely mapped to mtDNA reference genome. The same procedure was taken by Ye *et al.* (PNAS, 2014), as they mentioned in their Methods section that “Only reads uniquely mapped to the mitochondrial genome were recorded to minimize the complications of NumtS”. In fact, we required that all reads included in the analysis have a Mapping Quality Score (MAPQ in alignment software bwa) >=20. MAPQ is the phred-scaled probability of the alignment being wrong, so that MAPQ > 20 means the theoretical probability of the alignment being wrong is less than 1%. Meanwhile, for non-uniquely mapped reads (i.e., reads that could be mapped to two places equally well), bwa would give a MAPQ score of 0. But this does not necessarily mean that no reads included in our analysis can be aligned to NUMTs; it is possible that some reads can still be aligned to NUMTs, but with considerably more mismatches compared to the alignment to mtDNA reference (we used bwa’s default of 5 maximum mismatches for reads with length around 100 bp at the alignment step). Therefore, such reads are very unlikely to be from NUMTs even though they can be mapped to NUMTs. To show this directly, we did realignment experiments with the mtDNA reads supporting heteroplasmies. We randomly picked 10 individuals (in total, 19 heteroplasmies) based on the order of their IDs among all the individuals with at least one heteroplasmy. For each identified heteroplasmy from each individual, we extracted the reads that cover the alternative or the reference allele at the heteroplasmic site and their corresponding paired end sequence. We independently mapped those paired-end reads using bwa to two references: 1) mtDNA reference + NUMTs, or 2) NUMTs only, while allowing a maximum of 5 mismatches. NUMT sequences (766 sequences) were obtained by downloading the hg19 NUMT genomic coordinates from the NUMT track of the University of California, Santa Cruz (UCSC) Genome Browser. When the reads were mapped to reference 1) (mtDNA reference + NUMTs), all the reads were aligned to mtDNA, which is reassuring. When the reads were mapped to reference 2) (NUMTs only), some reads were unmappable and others were mapped to one NUMT with a large number of mismatches. By comparing the number of mismatches between the alignment to mtDNA and that to the NUMT, we calculated the **E**xtra **M**is-**M**atches (**EMM** = #mismatches for the best alignment to NUMTs - #mismatches for the alignment to mtDNA for a pair of reads). We then classified reads supporting heteroplasmies into three categories: a) unmappable to NUMTs or with EMM>=3 (those are reads with “strong evidence” of coming from mtDNA); b) with EMM = 2 (those are reads with “moderate evidence” of coming from mtDNA); c) with EMM = 1 (those are reads with “weak evidence” of coming from mtDNA).

The results for the 19 heteroplasmies are now summarized in Supplementary Table S5. In the vast majority of cases, we have a large number of reads that are in Categories a) and b) and have almost no reads from Category c), showing strong evidence that those reads are highly unlikely to have come from NUMTs -- therefore the heteroplasmies are real. In fact, among the 2,912 reads we investigated that support the 19 heteroplasmies, 84.4% of reads are in the “strong evidence” Category, 8.5% of reads are in the “moderate evidence” Category, and only 7.1% of reads are in the “weak evidence” Category. There are two cases that merit further discussion (heteroplasmies at position 6,750 for subject 36460, and at position 8,838 for subject 5655). In both cases, we observe fair numbers of reads with “weak evidence”; however, even if we exclude those reads, we still have substantial number of reads with “strong” or “moderate” evidence to support the heteroplasmy. We further note that even for reads in the “weak evidence” Category, they are still more likely to be from mtDNA than from NUMTs. Of course, excluding the reads with “weak evidence” could affect the estimates of minor allele fractions (i.e., heteroplasmy levels), but would not put in doubt the existence of those heteroplasmies.

In addition to including only reads with read-level mapping quality (MAPQ) >=20 in the analysis, we believe the following four factors together also minimize any impact of NUMTs on heteroplasmy identification: 1) the copy number of mtDNA is far higher than that of NUMTs, and we required that the minor allele fraction (MAF) for a heteroplasmy achieve a value ≥ 4.0%; 2) sequencing was done for paired ends; 3) we considered only reads with base Quality Score (QS) > 20 at the position of interest; and 4) we required that all alleles of a heteroplasmy are observed at least once in both forward and reverse strand sequence reads.

In summary, the preponderance of reads, including those predicting heteroplasmies are unambiguously assigned to mtDNA, and a limited number of reads are comparably probable for mtDNA or NUMTs. But in any case, even if some reads from an NUMT are falsely mapped to mtDNA, given that the copy number of mtDNA is far higher than that of nuclear DNA, they would have little effect on the identification of heteroplasmies because they would rarely meet the 4% MAF threshold we have set of declaring heteroplasmy. Furthermore, for any projected applications of this method, standard sequencing reads now have reached lengths at least 50% longer than the 100 bp average in the sample analyzed here, so that deviation of mtDNA sequences from NUMT sequences would be even greater.

NUMTs could also affect the estimation of mtDNA copy number. However, we believe that the impact on mtDNA copy number estimation and on downstream analyses is also minimal. First, as we showed in the “realignment” experiment, the vast majority of reads included in our analysis should be from mtDNA. Second, we carried out Q-PCR experimental validation for 18 randomly chosen samples and showed that the computational estimates and experimental measurements for copy number are in a similar range (Supplementary Figure S6), with a relatively high correlation of 0.82. Third, we included one extra safeguard in all the downstream analyses (e.g., heritability analysis): we performed an inverse-normal transformation for mtDNA copy numbers (i.e., assigning quantiles in a normal distribution to mtDNA copy numbers based on their ranks). Even if NUMTs had some impact on copy number estimates, their impact is likely to be similar for all the individuals, and hence the ranks of the estimates should remain unchanged. Therefore, the downstream analyses based on ranks rather than real values of the copy number estimates are very unlikely to be influenced by NUMTs.

**6. qPCR method for the experimental validation of mtDNA copy number estimation**

The NovaQUANT Human Mitochondrial to Nuclear DNA Ratio Kit compares the levels of nuclear to mitochondrial DNA (mtDNA) in a human DNA sample and hence estimates the mtDNA copy number. It uses qPCR as a quantitative assay to measure 4 different genes: 2 mitochondrial genes (ND1 and ND6) and 2 nuclear genes (BECN1 and NEB). Ct (cycle threshold) values obtained from qPCR are used to represent the level of each gene, and the average difference of Ct values between two nuclear genes and two mitochondrial genes can be directly used to calculate the mtDNA/nuclear DNA copy number ratio.

**7. Selection of unrelated individuals from the whole sequenced cohort**

We have pruned the dataset of 2,077 whole genome sequenced individuals for relatedness. Specifically, we computed the genome-wide proportion of pairwise identity by descent sharing (pi_hat) based on a random subset of 1 million common SNPs (MAF in 1000 Genomes population > 5%). For each pair of individuals with pi_hat > 0.07, we preferentially removed the offspring if in a trio, or otherwise the individual appearing to be more related to the rest of the sample (by the sum of pi_hat in all other relationships with pi_hat > 0.07), until there are no relationships with pi_hat > 0.07 left. In total, this removed 1,493 individuals, leaving 584 unrelated individuals for analysis.

**8. Further discussion on inheritance of heteroplasmies in trios**

The 66 inherited heteroplasmies all have the same genotypes in the corresponding children and their mothers, which is reassuring. However, they don’t necessarily have the same minor alleles. Supplementary Figure S9 is the scatterplot of Alternative Allele Fractions (AAF, the allele fraction for the non-reference allele) in children and their mothers. Using the two blue lines (vertical and horizontal lines at AAF=0.5) to divide the region into four quadrants, points in the upper left and lower right quadrants represent heteroplasmies with flipped minor alleles (for example, those in the upper left quadrant have the alternative alleles as minor alleles in a mother but have the reference alleles as minor alleles in a child). Indeed, among 66 inherited heteroplasmies, 20 (30.3%) heteroplasmies have different minor alleles in children and their mothers. This observation reflects both the “bottleneck” theory about passage of mtDNA variants from mothers to children and drift during the many generations of white cells in the mothers and children before sampling.

In addition to the 66 inherited heteroplasmies, there are 141 heteroplasmies identified in children but not seen in their mothers’ DNA (non-inherited heteroplasmies). Their MAFs range from 0.04 (since the MAF cut-off was set at 0.04) to 0.47. Supplementary Figure S10 shows the histograms of MAFs in children for inherited and non-inherited heteroplasmies. Compared to inherited heteroplasmies, non-inherited heteroplasmies generally have significantly lower minor allele fractions, consistent with the possibilities that many of them are newly formed.

With regard to false negatives, we again looked at the 141 heteroplasmies in children that were not identified as heteroplasmies in mothers. 103 sites (73%) had no evidence of heteroplasmy in mothers; 38 sites (27%) had two alleles (and in all cases they were the same two alleles seen in the children), but the MAF did not pass the 4% threshold [19 sites (13.5%) had MAF < 2% and 19 sites (13.5%) had MAF between 2% and 4%]. Considering that the mothers had the same two alleles as the children at the 38 sites, some of these sites could be heteroplasmic sites with low MAFs, but with the current data, we can not differentiate them clearly from sequencing errors.

Studying the inheritance of multiple heteroplasmies, we again looked at the data of 333 trios: 44 (13.2%) mothers have more than one heteroplasmy. The majority of them did not pass all the heteroplasmies to their children; in only 6 cases did mothers pass all their heteroplasmies to their children. This again supports the “bottleneck” theory and variable drift as possibilities.
